# Office‐Based Multilevel Radiofrequency Ablation for Mild‐to‐Moderate Obstructive Sleep Apnea

**DOI:** 10.1002/oto2.19

**Published:** 2023-02-17

**Authors:** Howard Herman, Jordan Stern, David M. Alessi, Keith A. Swartz, Marion Boyd Gillespie

**Affiliations:** ^1^ ENT of Georgia Atlanta Georgia USA; ^2^ Blue Sleep Center New York City New York USA; ^3^ Alessi Clinic Beverly Hills California USA; ^4^ AOO ENT Specialists of the Rockies Denver Colorado USA; ^5^ Department of Otolaryngology‐Head and Neck Surgery University of Tennessee Health Science Center (UTHSC) Memphis Tennessee USA

**Keywords:** obstructive sleep apnea, radiofrequency, radiofrequency ablation

## Abstract

**Objective:**

Investigate multilevel radiofrequency ablation (RFA) as an alternative therapy for patients with mild‐to‐moderate obstructive sleep apnea (OSA).

**Study Design:**

Prospective, open‐label, single‐arm, nonrandomized clinical trial.

**Setting:**

Multicenter academic and private clinics.

**Methods:**

Patients with mild‐to‐moderate OSA (apnea‐hypopnea index [AHI] 10‐30; body mass index ≤ 32) were treated with 3 sessions of office‐based RFA to the soft palate and tongue base. The primary outcome was a change in the AHI and oxygen desaturation index (ODI 4%). Secondary outcomes included subjective sleepiness level; snoring level; and sleep‐related quality of life.

**Results:**

Fifty‐six patients were enrolled, with 43 (77%) completing the study protocol. Following 3 sessions of office‐based RFA to the palate and base of the tongue, the mean AHI decreased from 19.7 to 9.9 (*p* = .001), while the mean ODI (4%) decreased from 12.8 to 8.4 (*p* = .005). Mean Epworth Sleepiness Scale scores declined from 11.2 (±5.4) to 6.0 (±3.5) (*p* = .001), while Functional Outcomes of Sleep Questionnaire scores improved from a mean of 14.9 at baseline to 17.4 (*p* = .001). The mean visual analog scale snoring scale was reduced from 5.3 (±1.4) at baseline to 3.4 (±1.6) at 6 months posttherapy (*p* = .001).

**Conclusion:**

Office‐based, multilevel RFA of the soft palate and base of the tongue is a safe and effective treatment option with minimal morbidity for properly selected patients with mild‐to‐moderate OSA who are intolerant or refuse continuous positive airway pressure therapy.

Obstructive sleep apnea syndrome (OSAS) causes repetitive episodes of upper airway obstruction during sleep, usually in association with a reduction in blood oxygen saturation.^1^ The prevalence of moderate‐to‐severe OSAS in the middle‐aged population is estimated to be up to 23% in women and 49% in men.^2^, ^3^ On the basis of these numbers, the global prevalence of sleep‐disordered breathing is estimated to be close to 1 billion people.^2^

Untreated mild‐to‐moderate obstructive sleep apnea (OSA) is associated with increased healthcare costs, motor vehicle accidents, and loss of work productivity.^4^, ^5^ First‐line treatment for many OSAS patients is nasal continuous positive airway pressure (CPAP). When used adequately, CPAP improves sleepiness, performance, quality of life, and cardiovascular risk,^6^, ^7^, ^8^ however 10% to 35% of patients fail to maintain CPAP use over time.^9^, ^10^, ^11^, ^12^, ^13^

Radiofrequency ablation (RFA) has demonstrated promise in reducing snoring and sleepiness symptoms.^14^ The procedure can be performed in the ambulatory setting under topical and local anesthesia. RFA has been applied as a second‐line treatment or adjunctive therapy with other sleep procedures for mild‐to‐moderate OSA if CPAP therapy is not adhered to or tolerated.^15^

The study's aim is to assess the treatment effect and safety of RFA in a cohort of non‐obese patients with mild‐to‐moderate OSAS. The study was specifically designed to address the effectiveness of multilevel (soft palate and tongue) treatment when applied over 3 treatment sessions.

## Methods

### Study Design and Objectives

This study is a multicenter, prospective, nonrandomized, Food and Drug Administration (FDA)‐approved study (NCT02349893) performed from 2017 to 2020. Institutional Review Board (IRB) approval was granted at each site by various IRBs including the University of Tennessee Health Science Center (M.B.G.); Solutions IRB, LLC (J.S.; H.H.; D.M.A.); and WCG IRB (K.A.S.). The device used in the study is the Celon ProSleep Plus (Olympus), a single‐prong bipolar radiofrequency applicator currently available and FDA‐approved within the United States for submucosal coagulation of the soft palate for the treatment of habitual snoring. The study's aim was to evaluate the safety of multilevel (soft palate and tongue base) RFA therapy for patients with mild‐to‐moderate OSA and to demonstrate the clinical effect 6 months after treatment. The study was funded by Olympus Winter & Ibe GmbH which covered study costs for participants and study sites. The study design was approved by the FDA to support an application for extended use in the base of the tongue region for patients with mild‐to‐moderate OSA. The full study protocol with detailed descriptions of results is available in Supplemental Appendix I, available online.

### Participants

Study participants were a cohort of adult patients (22 years and above) with mild‐to‐moderate OSA (apnea‐hypopnea index [AHI] 10‐30) and a body mass index (BMI) ≤ 32 kg/m^2^; intolerance or inadequate adherence to CPAP; self‐report of daytime somnolence; evidence of narrowing of the airway at the level of the soft palate and tongue base on supine fiberoptic examination; no prior surgical treatment for OSAS other than nasal surgery or tonsillectomy; and a regular nightly sleep partner. Exclusion criteria included comorbid sleep disorders; tonsillar hypertrophy (Brodsky 3‐4+); nasal or supraglottic obstruction on examination; ASA Classes III‐V; and drug or alcohol abuse or current participation in another research study. An AHI range of 10 to 30 is selected to conform with the Sher criteria which define surgical success as a 50% reduction in AHI and an overall AHI <20. There was concern that patients with baseline AHI between 5 and 10 could be considered unsuccessful even if their overall AHI was normalized <5 following treatment.

Screening studies included home WatchPAT 200S‐3 for diagnosis of OSAS or full in‐laboratory polysomnography (PSG; performed within 12 months of study enrollment). Although the WatchPAT device may underestimate the severity of OSA, screening with the device was considered acceptable since it would be followed by a full‐night baseline PSG. Following informed consent, all subjects underwent a subsequent baseline in‐laboratory PSG unless a full in‐laboratory PSG within 6 months was available. PSGs were scored using American Academy of Sleep Medicine criteria for apnea (>90% reduction in peak thermal sensor from baseline for ≥10 seconds) and hypopnea (≥50% reduction in baseline nasal pressure signal for ≥10 seconds with either ≥3% desaturation event or associated arousal).

#### Intervention

Participants underwent 3 radiofrequency treatments (4‐6 weeks apart) in an outpatient setting. Topical Cetacaine (benzocaine 14.0%; butamben 2.0%; and tetracaine hydroclhloride 2.0%) or HurriCaine (20% benzocaine) spray was applied to mucosal surfaces, followed by injection of 5 to 8 cc of 1% lidocaine with 1:100,000 epinephrine to both the body of the soft palate and the dorsal tongue at the level of the circumvallate papilla.

**Figure 1:**
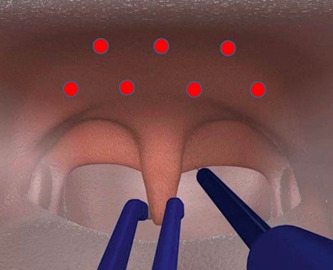
Diagram of approximate treatment sites on the soft palate.

*Soft palate RFA treatment*: A single‐prong RFA applicator (CelonProSleep Plus; Olympus) was used to create 7 lesions of 54 joule (J) each (Celon Power Setting 12 W) in a prescribed pattern (Figure 1).

**Figure 2:**
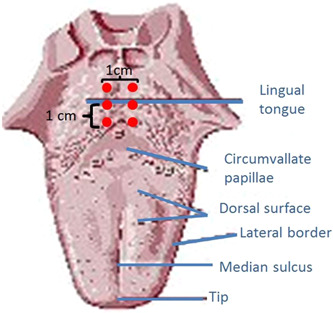
Diagram of the approximate treatment sites on the base of the tongue.

*Tongue base RFA treatment*: Using the same single‐prong RFA applicator, each patient then underwent 6 lesions of RFA treatment (CelonProSleep plus single‐prong applicator 80‐84 J each) to the base of the tongue using the prescribed pattern (Figure 2).

#### Postprocedure Care

Patients were monitored for 3 hours following treatment. In addition, participants were provided with prescriptions for an antibiotic (amoxicillin or clindamycin), oral steroid for 7 days (methylprednisolone taper pack), and oral pain medication (hydrocodone/acetominophen 5/325 mg) to be taken as needed. Patient follow‐up occurred on Days 1, 3, and 10 posttreatment. At the follow‐up visits, patients completed the pain, speech, and swallowing visual analog scale (VAS) scale and a clinical examination was performed. The use of pain medication (hydrocodone‐acetaminophen 5/325 mg; maximum 2 tablets every 6 hours or 8 tablets per day; Narco®) in the first 7 days following the procedure was recorded. Adverse events (AEs) were reviewed and coded by severity and attributed to either the surgical procedure or the device.

#### Outcome Measures

The study's endpoint was to demonstrate a clinically significant reduction of OSA symptoms by showing an adequate reduction in AHI determined by PSG results 6 months after treatment. Treatment response was defined as a ≥50% reduction in the baseline AHI and an overall AHI <20.

Secondary endpoints included change in the Epworth Sleepiness Scale (ESS); VAS of speech and swallowing; the Functional Outcomes of Sleep Questionnaire (FOSQ) (score range is 5‐20 where a higher score indicates higher activity level); and a drowsiness in the past week VAS (score range, 0‐100; 0‐9 represents minimal drowsiness; 10‐39 represents mild drowsiness; 40‐69 represents moderate drowsiness; and 70‐100 represents significant drowsiness). The Bed Partner Questionnaire was completed by the regular bed partner (score range is 0‐10, while 0‐3 means no snoring problem; 4‐6 mild snoring; 7‐9 moderate snoring; and 10 represents severe snoring disturbance).

#### Statistical Analysis

An a priori power analysis estimated a minimum of 34 subjects were needed to complete the study to demonstrate study endpoints with an anticipated dropout rate of 10 with an enrollment goal of at least 8 subjects per study site. Data were analyzed using the statistical software R: A Language and Environment for Statistical Computing, R Core Team, R Foundation for Statistical Computing, 2021 Version 4.05 2021. For continuous parameters, descriptive statistics including mean, standard deviation, median, and range are reported. For ordinal parameters, counts and percentages are reported in addition to the mean, standard deviation, median, and range.

The statistical analysis of the PSG data at baseline and follow‐up visits was analyzed using paired *t* test and linear‐by‐linear *χ* ^2^ tests for the AHI levels. Comparison of questionnaires' scores (baseline vs follow‐up visits) was tested using paired *t* test. Testing of the repeated measurements is carried out with a mixed model with random intercept using nlme package. All statistical tests were performed at a significance level of 0.05, with no corrections for multiple testing.

## Results

Fifty‐six patients were recruited for the study with 43 patients completing the protocol. Thirteen dropouts included 12 patients who were lost to follow‐up and 1 patient with an unreadable final PSG due to device failure. This patient refused to undergo a repeat PSG. The baseline characteristics of study participants are shown in Table 1. In general, subjects were middle‐aged overweight men demonstrating OSAS‐related quality of life deficits and excessive daytime somnolence. Of the 43 patients who completed the protocol, 7 (16%) had a prior tonsillectomy and 7 (16%) had undergone a previous septoplasty.

**Table 1:** Baseline Characteristics of Patients Completing the Study (N = 43)

Baseline characteristic	Normative value	Subject value
Gender (% male)		30/43 (70)
Mean age, y (SD)		50.7 (11.2)
Mean body mass index, kg/m^2^ (SD)	<25	27.2 (3.7)
Mean apnea‐hypopnea index (events/hour sleep time) (SD)	<5	19.7 (7.1)
Mean oxygen desaturation index, 4% (SD)	< 5	12.8 (7.7)
Mean lowest O_2_ saturation (%) (SD)	>90%	84.2 (5.3)
Mean Epworth Sleepiness Scale score (SD)	<10	11.2 (5.4)
Mean snoring VAS score (SD)	0	5.3 (1.3)
Mean bed partner snoring VAS score (SD)	0‐3	6.9 (2.2)
Mean functional outcomes of sleep Questionnaire (FOSQ) (SD)	>17.8	14.9 (4.4)
Mean drowsiness in the past week VAS score (SD)	<10	54.3 (26.3)

Abbreviations: FOSQ, Functional Outcome of Sleep Questionnaire; SD, standard deviation; VAS, visual analog scale.

### Primary Outcome Measures

The primary outcome measure of AHI change from baseline to 6 months postintervention is summarized in Table 2. Overall, 22/43 (51%) subjects were considered complete responders with a ≥50% reduction in baseline AHI and an overall AHI <20 at study completion. Whereas 25 patients (58%) had AHI scores below 20 at baseline, 39/43 (91%) had scores below 20 following treatment. A statistically significant reduction in AHI (*p* = .001) was observed at 6 months follow‐up.

**Table 2:** Primary AHI Outcomes at Baseline and 6 Months Post‐RFA Treatment (N = 43)

Outcome measure	Baseline	6‐mo posttreatment	*p* value
Responder (AHI reduction ≥50; overall AHI <20) (%)		22/43 (51)	
Normal (AHI <5) (%)	0 (0)	16 (37)	
Mild OSA (AHI 5‐15) (%)	16 (37)	16 (37)	
Moderate OSA (AHI 15‐30) (%)	27 (63)	11 (26)	
Mean AHI (±SD)^a^	19.70 (7.10)	9.86 (8.28)	.001
Median AHI (range)^b^	17.80 (10.40‐34.90)	7.5 (0.00‐35.90)	.001

Abbreviations: AHI, apnea‐hypopnea index; OSA, obstructive sleep apnea; RFA, radiofrequency ablation; SD, standard deviation.

^a^ Calculated with paired *T* test.

^b^ Wilcoxon signed rank test.

Subgroup analysis was performed on 27/43 (63%) of subjects with moderate OSA (AHI >15‐30) and 16/43 (37%) with mild OSA (AHI 10‐15) on the screening WatchPAT examination. The baseline AHI results of the PSG in the moderate group ranged from 15.9 to 34.9 and included 4 patients with AHI >30 (range, 30‐34.9) who were found to have severe OSA on baseline PSG. A total of 15/27 (56%) of the moderate group demonstrated a 50% reduction of AHI with an overall AHI <20 at study completion with a mean AHI reduction of −13.1 (*p* = .0000023) from baseline mean of 23.7 (±5.9) to final mean of 10.7 (±9.6). The AHI results of the mild group (baseline AHI; range, 11‐14.9) demonstrated 8/16 (50%) with a 50% reduction in final AHI with a mean AHI reduction of −4.41 (*p* = .009) from a baseline mean of 12.9 (±1.4) to 8.5 (±5.5).

Oxygen desaturation index (ODI; 4% desaturation) results are shown in Table 3. Eleven (26%) patients had incomplete ODI scores due to the inability to obtain the baseline ODI from the historical PSG sleep study performed within 6 months of enrollment. Three of these 11 patients did not have ODI scored at the 6‐month follow‐up PSG. Overall, 23/32 (72%) demonstrated ODI reduction following treatment with a mean ODI reduction of 33% (*p* = .006). An ODI reduction of ≥50% was noted in 16/32 (50%) of subjects with complete data.

**Table 3:** Primary Outcome ODI (4%) Outcomes at Baseline and 6 Months Post‐RFA Treatment (N = 32)

Outcome measure	Baseline	6‐mo posttreatment	*p* value
ODI 4 reduction ≥25%		23/32 (72%)	
Mean ODI (±SD)^a^	12.79 (7.74)	8.36 (8.74)	.006
Median ODI (range)^b^	11.65 (0.00‐31.20)	6.32 (0.00‐30.40)	.008

Abbreviations: ODI, oxygen desaturation index; RFA, radiofrequency ablation; SD, standard deviation.

^a^ Calculated with paired *t* test.

^b^ Wilcoxon signed rank test.

The 21 (49%) treatment nonresponders were offered standard of care management of their OSA following the completion of the study including CPAP, oral appliance therapy, and/or additional surgical procedures. This care was outside of the study and was not included as part of the study data.

### Secondary Outcome Measures

All self‐reported questionnaire responses at the 6‐month follow‐up visit demonstrated statistically significant improvement compared to baseline (Table 4). Based on the bed partner report, intrusive snoring (very intense snoring or bed partner leaving the room) was reduced from 63% at baseline to 7% at 6 months follow‐up. The percentage of participants who reported normal ESS scores (<10) increased from 67% at baseline to 88% at 6 months posttreatment with a reduction of 5.6 points on average. At baseline, only 14% reported a normal FOSQ score (>17.9) but increased to 58% at 6 months posttreatment with an average increase of 2.5 points. In addition, subjects endorsed a 43% mean reduction in drowsiness over the prior week.

**Table 4:** Mean Values of Self‐Reported Items (N = 43)

Item	Baseline mean (SD)	6‐mo posttreatment mean (SD)	*p* value (paired *T* test)
Snoring VAS	5.33 (1.34)	3.41 (1.66)	.001
Bed partner snoring VAS	7 (2.16)	3.12 (2.38)	.001
Epworth Sleepiness Scale	11.19 (5.40)	5.95 (3.51)	.001
Functional Outcome of Sleep Questionnaire	14.91 (4.43)	17.41 (2.17)	.001
Drowsiness in the past week VAS	54.34 (26.33)	31.08 (24.72)	.001

Abbreviations: SD, standard deviation; VAS, visual analog scale.

### AEs

No serious AEs were observed during the study. Eleven AEs were reported in 5 (12%) patients. One patient (2%) had mild dysphagia that resolved after 3 days. Five patients (9%) had tissue edema with or without mucosal ulceration treated with saline gargles or oral antibiotics. Two of the patients received an additional oral steroid dose pack.

Pain level was documented using the VAS scale (0‐10, where “0” indicates no pain and “10” indicates unbearable pain). The average pain level immediately after RFA treatment was 1.7 (±1.1) and decreased to a mean of 0.29 (0.72) by Day 7. In addition to the low levels of pain, the vast majority of study patients reported complete recovery of the soft palate and the tongue base at 6 weeks after the RFA treatment (95% and 97%, respectively) and 100% at 6 months.

## Discussion

OSAS is a prevalent disorder estimated to be present in 10% to 17% of adult men and 3% to 9% of adult women.^16^ This translates into approximately 13 million individuals over the age of 30 within the United States.^17^ The burden of the disorder increases with age with a prevalence of 50% in age groups older than 65 years.^17^ Untreated OSAS is associated with an increased risk of cardiovascular disease, cerebrovascular disease, hypertension, perioperative complications, and premature all‐cause mortality.^18^ Untreated OSAS causes a profound reduction in quality of life due to symptoms of snoring, poor sleep quality, and daytime sleepiness. CPAP is the recognized first‐line therapy for OSAS, however, up to 40% to 50% of patients fail to adhere to the recommended use of 4 or more hours of therapy per night. With regard to mild‐to‐moderate OSA, it is estimated that up to 1 in 5 normal‐weight adults (BMI 25‐28) in the United States has mild OSA, and 1 in 15 has at least moderate OSA.^19^ Therefore, there is a recognized need for alternative therapies to meet this public health challenge.

Non‐CPAP treatment options for moderate OSA (AHI <30) include mandibular advancement devices (MAD) and/or various surgical interventions. MADs are effective for mild‐to‐moderate OSA but may not be acceptable to all patients due to cost; the need for nightly use; and side effects such as drooling, temporomandibular joint discomfort, tooth pain, and xerostomia.^20^ Surgical interventions are effective in select patients but have variable outcomes, involving expense, anesthesia risk, and the potential for bleeding, infection, pain, and poor wound healing. The ideal treatment is affordable, device‐free, and office‐based with minimal side effects that effectively reduce snoring and daytime sleepiness.

RFA has been used as a potentially less morbid approach to stiffen and provide structural support to collapsible upper airway segments. Radiofrequency energy causes tissue ions to become agitated due to changes in electrical flow inherent in alternating current at relatively low temperatures (60‐95°C).^21^ The lesion created by RFA creates protein coagulation and results in congestion, edema, and an acute inflammatory response within the first 24 hours. Over a period of 72 hours, the treated area creates focal necrosis which is transformed into fibrotic tissue over the course of 10 days.^21^

A variety of FDA‐cleared RFA devices have demonstrated promise as a treatment alternative for OSA in multiple published studies.^22^, ^23^, ^24^, ^25^ In these studies, repeated RFA of the soft palate and base of the tongue region resulted in reductions in AHI and daytime sleepiness without significant complications. RFA has several advantages over traditional surgical approaches including its ability to address multiple levels of the airway (nose, palate, and tongue); its ability to perform in the office under local anesthesia; lower cost; and minimal pain and morbidity.

The present trial was designed to maximize the above advantages of RFA for a cohort of patients most likely to demonstrate benefit, namely nonobese patients with symptomatic mild‐to‐moderate OSAS. Based on the prior literature, it is clear that RFA is most effective as a treatment is used to treat multilevel sites of collapse (soft palate and tongue) in a repeated fashion designed to allow sufficient volumetric tissue reduction and fibrosis to occur. The results of this study demonstrate that repeated application of RFA energy to multiple sites of airway collapse in appropriately selected patients results in a significant reduction in AHI with improvement in snoring and daytime sleepiness with a low level of patient morbidity.

Limitations of the study are mainly due to the nonrandomized design without placebo control. Although subjective patient questionnaires are subject to the placebo effect, the primary study outcomes of AHI and ODI (4%) were based on objective testing criteria. Thirteen (23%) patients were lost to follow‐up and, therefore, it is difficult to know if their outcomes would be consistent with the group that completed the entire course of therapy. It is reasonable to assume that some patients may have difficulty tolerating a course of care that requires several invasive treatment sessions over a 3‐ to 4‐month period. The study endpoint at 6 months does not allow projection of long‐term results which will require further follow‐up in order to determine the length of treatment effect. In addition, the study cohort was limited to patients with mild‐to‐moderate OSA (AHI 10‐30) with BMI ≤32 kg/m^2^ and therefore similar results cannot be assumed in patients with more severe OSAS who may benefit from other currently available treatment options.

## Conclusions

This study evaluated the safety and effectiveness of RFA ablation treatment in the base of the tongue and soft palate for improving mild‐to‐moderate OSAS. The study found that RFA provides significant improvements in PSG measures of OSA and clinically meaningful improvements in patient self‐reported outcomes. The therapy had few side effects and was well‐tolerated with a low level of pain and morbidity.

## Author Contributions

**Howard Herman**, site PI, subject enrollment, data acquisition, data analysis, manuscript preparation, and review; **Jordan Stern**, site PI, subject enrollment, data acquisition, data analysis, manuscript preparation and review; **David M. Alessi**, site PI, subject enrollment, data acquisition, data analysis, manuscript preparation and review; **Keith A. Swartz**, site PI, subject enrollment, data acquisition, data analysis, manuscript preparation and review; **Marion Boyd Gillespie**, site PI, subject enrollment, data acquisition, data analysis, manuscript preparation and review; research presentation.

## Disclosures

### Competing interests

None.

### Sponsorships

Olympus Winter & Ibe GmbH.

### Funding source

This article was supported by Olympus Winter & Ibe GmbH, Hamburg, Germany (Institutional Review Board fees; compensation for study coordinator; surgeon; statistician; subject incentive; disposable devices; and equipment loan).

## Supporting information

Final Clinical Investigation Report submitted to U.S. Food and Drug Administration (FDA).
