# Supplementary material for: Office‐Based Multilevel Radiofrequency Ablation for Mild‐to‐Moderate Obstructive Sleep Apnea
Source: OTO Open. 2023 Feb 17;7(1):e19. doi: 10.1002/oto2.19 (PMC10046721; doi:10.1002/oto2.19)
Supplement: Supplementary file 1 — Final Clinical Investigation Report submitted to U.S. Food and Drug Administration (FDA). [file OTO2-7-e19-s001.docx]

Clinical Investigation Report

**Radiofrequency Ablation for Multi-level Obstructive Sleep Apnea: A Single-arm, Multicenter study**

**Study design:** Multi center, Open label, single arm, non-randomized study

**Study Duration:** The duration of the study is 24 weeks, and follow-up data continue to be collected for 6 months post-treatment

**Study population:** Mild to moderate Obstructive Sleep Apnea Syndrome (OSAS) patients (AHI of 10-30 and BMI ≤ 32***)***

Investigational

Devices: CelonProSleep *plus* RF electrode (Olympus Winter & Ibe, Hamburg,

Germany

**SPONSOR:** Olympus Winter & Ibe

Kuehnstr. 61

22045 Hamburg

Germany

**Sponsor Representative**: Dr. Samuel Faran

CIP identification No.: 990211

Publicly accessible database registration number: ClinicalTrial.Gov registration number - NCT02349893

Coordinating investigator: Prof. M. Boyd Gillespie

Biostatistician: Dr. Diklah Geva, Integristat

CRO: Dr. Hanna Levy, Qsite Medical

This clinical study was conducted in compliance with the declaration of Helsinki, good clinical practice (GCP), ISO 14155:2020, and all applicable national laws and regulations.

**CONFIDENTIALITY STATEMENT**

This clinical investigation report contains confidential information and all information and data contained within this document are the property of Olympus. It shall not be reproduced or copied in whole or in part, nor shall the contents be disclosed by any mean without prior written permission from Olympus, nor shall anyone make any use of it that is contrary to the expressed or implied wishes of Olympus.

Date of report:

22^nd^ December 2021

Author(s) of report: Dr. Hanna Levy, Qsite Medical

Table of content

[1. Summary 6](#_Toc92841037)

[2. Introduction 11](#_Toc92841039)

[2.1 Obstructive Sleep Apnea 11](#_Toc92841040)

[2.2 Diagnosis of Obstructive Sleep Apnea 11](#_Toc92841041)

[2.3 Surgical Therapy for OSA 12](#_Toc92841042)

[2.4 CelonProSleep plus 14](#_Toc92841043)

[2.5 Safety of RF ablation of the soft palate and base of tongue 15](#_Toc92841044)

[3. Investigational device and methods 17](#_Toc92841045)

[3.1 Investigational device description 17](#_Toc92841046)

[3.1.1 Intended use of the investigational device 17](#_Toc92841047)

[3.1.2 Any changes to the investigational device during the clinical investigation or any changes from the IB 17](#_Toc92841048)

[3.2 Clinical investigation plan (CIP) 18](#_Toc92841049)

[3.2.1 Objective 18](#_Toc92841050)

[3.2.2 Design 19](#_Toc92841054)

[3.2.3 Ethical considerations 19](#_Toc92841055)

[3.2.4 Data quality assurance 19](#_Toc92841056)

[3.2.5 Subject population 19](#_Toc92841057)

[3.2.6 Treatment and treatment allocation 21](#_Toc92841058)

[3.2.7 Concomitant medications/treatments 21](#_Toc92841060)

[3.2.8 Duration of follow-up 21](#_Toc92841061)

[3.2.9 Statistical design 22](#_Toc92841062)

[3.2.9.1Clinical investigation hypothesis or pass/fail criteria 22](#_Toc92841063)

[3.2.9.2 Sample size calculation 22](#_Toc92841064)

[3.2.9.3 Statistical Analysis Methods 23](#_Toc92841065)

[4 Results 24](#_Toc92841066)

[4.1 Clinical Investigation Initiating date 24](#_Toc92841067)

[4.2 Clinical Investigation completion date 24](#_Toc92841068)

[4.3 Disposition of subjects; number screened patients and patients who received therapy 25](#_Toc92841069)

[4.4 Subject demographics and other relevant baseline characteristics 26](#_Toc92841070)

[4.5 CIP Compliance 30](#_Toc92841071)

[4.6 Results of the primary endpoints 31](#_Toc92841072)

[4.6.1Analysis of AHI Results (AHI reduction) 31](#_Toc92841073)

[4.6.2 Analysis of ODI Results (ODI reduction) 33](#_Toc92841075)

[4.6.3 Summary of Endpoint Results 34](#_Toc92841076)

[4.6.4 Analysis of OSA Results 35](#_Toc92841077)

[4.6.5 Analysis of Questionnaires’ Results 37](#_Toc92841078)

[4.6.6 Evaluation of pain level and recovery rate after RFA treatments 41](#_Toc92841085)

[4.6.7 Summary of all adverse events 42](#_Toc92841086)

[4.6.8 Adverse device effects 43](#_Toc92841087)

[4.6.9 Any needed subgroup analyses for special populations (i.e., gender, racial/cultural/ethnic subgroups), as appropriate 43](#_Toc92841088)

[4.6.10 An accountability of all subjects with a description of how missing data or deviation(s) were dealt with in the analysis, including subjects 43](#_Toc92841089)

[4.6.11 Listings of deaths and reasons for deaths 44](#_Toc92841090)

[5 Discussion and overall conclusions 45](#_Toc92841091)

[5.1Safety 45](#_Toc92841092)

[5.2 Efficacy of the RFA Treatment 45](#_Toc92841093)

[5.3 Clinical effect of RFA treatments on OSAS patients 46](#_Toc92841094)

[5.4 Questionnaire results 48](#_Toc92841095)

[5.5 Assessment of risks and benefits 48](#_Toc92841096)

[5.5.1 Any specific benefits or special precautions required for individual subjects or groups considered to be at risk 49](#_Toc92841097)

[5.5.2 Any limitations of the clinical investigation including but not limited to: 49](#_Toc92841098)

[5.5.3 Selection, retention, adherence (to CIP, instructions for use and the requirements of this document) of investigation sites and users, and investigation site environment type(s) 49](#_Toc92841099)

[5.5.4 Bias introduced by missing observations, by confounders and by 1) and 2) above 49](#_Toc92841100)

[6 Abbreviated terms and definitions 50](#_Toc92841101)

[7 Ethics 52](#_Toc92841102)

[7.2 Statement that informed consent was obtained and when it was obtained 52](#_Toc92841103)

[8 Investigators and administrative structure of clinical investigation 53](#_Toc92841104)

[8.1.1A list of investigators, including their affiliations 53](#_Toc92841105)

[8.1.2Names and addresses of any external organizations (such as core laboratories, CROs, consultants or other contractors) that contributed to the clinical investigation 53](#_Toc92841106)

[Annexes to the report 54](#_Toc92841108)

[Annex 1 Instructions for use 54](#_Toc92841109)

[Annex 2 Adverse Events 56](#_Toc92841110)

Annex 3 Patients AHI and ODI variables………………………………………………………………….58

Annex 4 Study Protocol……………………………………………………………………………………..60

[REFERENCES 116](#_Toc92841113)

# Summary

Title of the clinical investigation:

**Radiofrequency Ablation for Multi-level Obstructive Sleep Apnea: A Single-arm, Multicenter study**

Short introduction:

FDA-cleared devices have demonstrated promise as a treatment alternative for obstructive sleep apnea in multiple published studies^29, 30, 31, 34^. In these studies, repeated radio frequency ablation (RFA) of the soft palate and base of tongue region resulted in significant reductions in Apnea Hypopnea Index (AHI) and daytime sleepiness without significant complications. RFA has several advantages over traditional surgical approaches including its ability to address multiple levels of the airway (nose, palate, and tongue), ability to perform in the office under local anesthesia, lower costs, minimal pain, and morbidity. The Celon ProSleep *plus*, the device used in this study, was already used in a few studies to treat soft palate and base of tongue in Obstructive Sleep Apnea Syndrome (OSAS) patients^32, 45, 46^.

Introduction:

The United States is currently experiencing an increase in the incidence and prevalence of obstructive sleep apnea (OSA). With prevalence in middle-aged adults of 2 to 4% of the population, untreated OSA increases the risk for cardiovascular disease, including hypertension and heart failure, daytime sleepiness, and increased risk of motor vehicle accidents.

The first line and most common treatment for OSA is continuous positive airway pressure (CPAP), utilized by an estimated 3 million Americans. CPAP is effective in reducing the AHI if used properly. However, the nasal and/or facial mask required for CPAP during sleep may lead to poor adherence to therapy. Published studies on CPAP have shown that only 58 to 80% of patients accept CPAP therapy^7-10^, with 65 to 90% exhibiting long-term adherence to CPAP, therefore 10-40% of patients fail to maintain CPAP use over time^11^. Additionally, many patients treated successfully with CPAP have low treatment satisfaction due to facial discomfort, nasal blockage, abdominal bloating, and loss of intimacy with their bed partner. CPAP variations such as auto-titrating CPAP, heated and humidified air, and bi-PAP (different pressures on inspiration and expiration) have failed to consistently improve patient adherence to therapy, indicating a significant unmet need for CPAP alternatives in patients who are not adherent to therapy.

Surgical therapy for OSA is less common than CPAP therapy. Surgical therapy is based in part on an anatomic assessment of the likely sites of obstruction. Anatomic analysis is most commonly performed with fiberoptic examination of the upper airway, with radiographic imaging reserved for cases of suspected craniofacial abnormality.

Radiofrequency ablation (RFA) of the upper airway using FDA-cleared devices has demonstrated promise as a treatment alternative for obstructive sleep apnea in multiple published studies. RFA enables the surgeon to direct the delivery of a specific amount of radiofrequency energy, measured in joules, to a specific site at a controlled temperature. This radiofrequency energy is delivered at relatively low power and voltage. Application of radiofrequency energy in this manner causes tissue ions to become agitated due to changes in electrical flow inherent in alternating current. These ionic shifts result in resistive heating by the tissue itself and in comparison to electrocautery the production of low temperatures (60° to 95°C). Protein, which denatures at temperatures in excess of 47°C, undergoes tissue coagulation along with surrounding stromal and vascular tissue. The lesion created by RFA is consistent with tissue coagulation and results in congestion, edema, and an acute inflammatory response within the first 24 hours. Over a period of 72 hours, the treated area progresses to tissue necrosis which may change to fibrotic tissue over the course of 10 days.

The CelonProSleep *plus* is a rigid, sterile, single-use, bipolar electrosurgical electrode. It is used in conjunction with the electrosurgical generator CelonLab ENT. The device is indicated for the ablation and coagulation of soft tissue in otorhinolaryngology surgery including submucosal tissue shrinkage and tissue coagulation in the uvula/soft palate for the treatment of snoring.

The study summarized in this report was designed for demonstrating the safety and efficacy of the CelonProSleep *plus* device for the treatment of mild to moderate Obstructive Sleep Apnea Syndrome (OSAS) patients.

**Study objective:** The primary effectiveness of the study is to demonstrate a clinically significant reduction of OSAS from Baseline PSG sleep study to the 6-Month follow-up PSG in adults (≥ 22 years) with obstructive sleep apnea (AHI 10-30) and BMI ≤ 32.

**Study population:** Mild to moderate OSAS patients (AHI of 10-30 and BMI ≤ 32***)***

**Study design:** Multi center, open label, single arm, non-randomized study

Summary of results of the clinical investigation

**Safety**

No serious adverse events (SAEs) were detected or reported during the study. Eleven adverse events (AEs) were reported in five patients (Annex 2). Most of these adverse events occurred in two patients only. All adverse events were mild. Six out of the eleven cases were solved without any treatment and 5 cases were treated with saline or antibiotics. Two patients were administered steroids.

Pain level was documented using the VAS scale (0 - 10, where “0” indicates no pain and “10” indicates unbearable pain). Measurements of pain level were performed directly after the RFA treatment as well as 1-, 3-, 7- and 10-days post-intervention. The average pain level directly after RFA treatment was low, decreased at three days following treatment and continued to decline on subsequent post-treatment visits as well. The low level of pain and its rapid resolution was observed after each RFA treatment.

In addition to the low levels of pain, the vast majority of study patients reported complete recovery of the soft palate and the tongue base at six weeks after the RFA treatment (95% and 97% respectively) and 100% at six months follow-up.

The results on pain level and tissue recovery clearly indicate that RFA treatments performed with CelonProSleep *plus* are safe with rapid recovery, and a low level of tolerable pain..

**RFA treatment efficacy**

The following endpoints assessed the effectiveness of the RFA treatment performed with CelonProSleep *plus*:

1. AHI score post-intervention <20.

2. Reduction of at least 50% in AHI score post-intervention compared to baseline.

3. Reduction of at least 25% in ODI score post-intervention compared to baseline.

4. AHI reduction ≥50% and a reduction of ODI ≥25% at the 6-month follow-up PSG.

Study success was defined as: at least 50% of study patients are defined as Responder.

**Study results:**

1. **AHI score (<20) post-intervention**

Out of 43 patients who completed the study, 39 patients (**90.7%)** met this endpoint. This result is well above the endpoint requirement (at least 50% of study patients). Thus, this endpoint was met.

1. **Reduction in AHI score (≥50%) post-intervention**

Out of 43 patients who completed the study, 23 patients (**53.5%)** showed a reduction in their AHI over the required 50%. Therefore, this endpoint was met as well.

1. **ODI reduction ≥25% post-intervention**

Out of 32 patients who had a full set of ODI scores, 21 patients (**65.67%)** had a reduced ODI greater than 25%. This result is above the endpoint requirement (at least 50% of study patients).

1. **AHI reduction ≥50% and a reduction of ODI ≥25% at the 6-month follow-up PSG**.

Out of 32 patients who had AHI and ODI scores before and after treatment, 14 patients (**43.7%)** met this endpoint. The result of the combined endpoints is below endpoint requirement (at least 50% of study patients). This result is due to the smaller group of patients (32) that had ODI scores at baseline.

In sum, the analyses of the endpoints showed that over 50% of study patients fully met three endpoints out of four, while the 4^th^ endpoint was met by 43.7% of study’ patients (2 patients below the requirement of meeting the success criterion).

#### Clinical effects of RFA treatment on OSAS patients

In order to examine the impact of the RFA treatments in OSAS patients with the CelonProSleep *plus* we evaluated ***the change in sleep quality and symptom reduction*** after the RFA treatment in comparison to baseline. Sleep quality and OSA symptomatic were determined based on the AHI scores (normal, AHI <5; mild sleep apnea, AHI = 5-15; and moderate sleep apnea, 15≤ AHI ≤30).

No patient was defined as ***"normal"*** (AHI ≤5) at baseline, but 16 patients (out of 43 – 37.2%) were classified as “normal” after the RFA treatment (AHI score <5). In addition, while 27 patients (63%) were classified at base line as "*moderate”* (15≤ AHI ≤30), only 11 patients (25.6%) were classified as “moderate” after the RFA treatment.

The comparisons of AHI scores post-intervention with AHI scores at baseline reveal the high clinical benefit of the RFA treatments on sleep quality and symptoms in OSA patients. Most impressive is the improvement in sleep quality of 12 patients (28%) who were defined as "moderate" at baseline and as "normal" after the three RFA treatments. An additional four patients (9.3%) were defined as "mild" at baseline and as "normal" after RFA treatments.

**Results of various sleep Questionnaires**

Five questionnaires were presented to study participants, including one questionnaire which was addressed to the bed partner of the patients.

The analyses of the questionnaires indicate improvement in snoring, daytime sleepiness, level of activity, and drowsiness of the patients after the RFA treatment. Comparisons of baseline scores with the scores at six weeks after RFA treatment revealed statistical significance which continued six months post-intervention. The results demonstrate the effect of the RFA treatments in improvement of various aspects of daily life.

**Study duration**

The study lasted 6.5 years. In this long period 56 patients were recruited to the study, and only 43 (out of 56 -76.7%) completed the entire study course. This long study duration indicates the difficult in recruiting subjects and keeping them in the study, and the effect of loss of study sites during the course of the investigation. Patients found the study protocol too complicated with too many study visits (altogether 14 visits - 8 main visits and 6 physical examination visits after the RFA treatments), and in addition two sleep lab visits. This is also the reason for a large percentage of dropouts (24%). The difficulty in recruiting and maintaining the patients in the study is a factor that affects the results of the study. Nevertheless – study results do indicate the significant impact of the RFA treatments on the sleep quality of study patients.

Conclusion:

This study clearly shows that the multi-modal RFA treatments of base of tongue and soft palate provided by the CelonProSleep *plus* ***significantly improve*** the sleep quality and symptoms in patients suffering from OSAS with complete resolution of the disorder in select patients. Moreover, this treatment is minimally invasive and results in a very low pain level. In addition, the results also indicate that the level of risk associated with this treatment is very low and avoids the risk of general anesthesia. There were only a few adverse events and no serious adverse events.

Date of the clinical investigation initiation: November 2014

Completion date of the clinical investigation: June 2021

# Introduction

## Obstructive Sleep Apnea

The United States is currently experiencing an increase in the incidence and prevalence of obstructive sleep apnea (OSA). With prevalence in middle-aged adults of 2 to 4% of the population, untreated OSA increases the risk for cardiovascular disease, including hypertension and heart failure, daytime sleepiness, and increased risk of motor vehicle accidents^1-6^.

Despite its prevalence and increased recognition as a cardiovascular risk factor, OSA remains largely under diagnosed. The standard test for diagnosis of OSA is polysomnography (PSG), which produces outputs on several physiological variables. The apnea-hypopnea index (AHI), expressed as the number of apneas/hypopneas per hour of sleep, is the most used variable to measure the severity of disease. An AHI of 5 or greater when associated with daytime sleepiness connotes a diagnosis of sleep apnea, an AHI between 5 and 14 is defined as mild disease, an AHI of 15 to 30 is moderate and an AHI greater than 30 is severe disease. The goal of treatment of OSA is improvement of AHI and other key variables (such as lowest oxygen saturation, LSAT), and improvement of patient symptoms and reduced cardiovascular and overall mortality.

The first line and most common treatment for OSA is continuous positive airway pressure (CPAP), utilized by an estimated 3 million Americans. CPAP is effective in reducing the AHI if used properly. However, the nasal and/or facial mask required for CPAP during sleep may lead to poor adherence to therapy. Published studies on CPAP have shown that only 58 to 80% of patients accept CPAP therapy^7-10^, with 65 to 90% exhibiting long-term adherence to CPAP, therefore 10-40% of patients fail to maintain CPAP use over time^11^. Additionally, many patients treated successfully with CPAP have low treatment satisfaction due to facial discomfort, nasal blockage, abdominal bloating, and loss of intimacy with their bed partner. CPAP variations such as auto-titrating CPAP, heated and humidified air, and bi-PAP (different pressures on inspiration and expiration) have failed to consistently improve patient adherence to therapy, indicating a significant unmet need for CPAP alternatives in patients who are not adherent to therapy^12^.

## Diagnosis of Obstructive Sleep Apnea

The gold standard for the diagnosis of OSA remains the attended overnight level I polysomnogram (PSG). PSG’s include electroencephalogram (EEG), electro-oculogram (EOG), electromyogram (EMG), electrocardiogram (ECG), oronasal airflow, thoracic and abdominal movement, oxygen saturation, snoring level, and body position^13^. However, PSG has many limitations including high cost, long waiting lists, limited availability, and the need for technical expertise to perform and interpret. In addition, many patients find the PSG equipment too cumbersome and may be reluctant to spend the night in the laboratory. Thus, it is not possible to perform PSG studies for all individuals suspected of having OSAS and waiting duration for PSG may exceed months to years^18^, resulting in patients who are waiting long time for an adequate therapy.

As a result of these factors single and multiple channel monitoring systems have been introduced to screen for OSA. One of these systems is the WatchPAT 200S-3 (Itamar Medical Inc., Franklin, MA, USA)^[[1]](#footnote-1)^, which is a level III (minimum of 4 physiologic channels) portable diagnostic sleep device that is worn on a patient’s wrist along with two self-adhesive finger probes. The device measures several parameters including pulse Oximetry, heart rate, wrist actigraphy (muscle twitches), body position, snoring, and peripheral arterial tonometry (PAT). The WatchPAT 200S-3 uses patented algorithms which interpret the physiologic measurements to detect the presence of sleep-disordered breathing. A major advantage to the system is its ease of use which can be easily applied by the patients in the comfort of their own bedroom, an environment that best reflects the pattern of their sleep habits. Increasingly, the public and third-party payers are requiring home sleep testing devices such as WatchPAT as the initial diagnostic for sleep-disordered breathing due to increased patient acceptance and reduced cost of care.

In 2007, the AASM published its clinical guidelines for the use of unattended portable monitors in the diagnosis of OSA in adult patients^16^. These guidelines state that “the Portable Monitor­ing Task Force of the American Academy of Sleep Medicine (AASM) makes the following recommendations: unattended portable monitor­ing (PM) for the diagnosis of obstructive sleep apnea (OSA) should be performed only in conjunction with a comprehensive sleep evaluation. On December 14, 2007 the CMS released its proposed decision for modification of NCD policy 240.41 pertaining to coverage of CPAP therapy for adult obstructive sleep apnea (OSA). The proposed modification allows for an initial 12-week period of CPAP coverage when OSA is diagnosed using both a clinical evaluation and PSG performed in the sleep laboratory or a clinical evaluation and unattend­ed home sleep studies using a Type II, III or IV device^17^**.**

With WatchPAT 200S-3, both RDI and AHI scores are highly reproducible, showing correlation between home and in-laboratory sleep studies^e.g.19-24^. This study will utilize WatchPAT 200S-3 for screening of the qualification of the patient to participate in the study regarding his/her AHI score. In addition, patients will undergo WatchPAT home sleep study at both follow-up visits for determining changes compared to the screening visit. In order to decrease the risk of overestimation of OSA variables with increasing severity of OSA the current study will include only mild to moderate patients.

## Surgical Therapy for OSA

Surgical therapy for OSA is less common than CPAP therapy. Fewer than 100,000 surgical treatments for OSA are performed in the United States annually, despite the fact that up to a third (700,000) of the two million people in the U.S. who start CPAP each year may ultimately fail to adhere to CPAP over the long-term. Surgery is less commonly used for a number of reasons foremost of which is the inconsistent success and high pain and morbidity of the most commonly performed procedure uvulopalatopharyngoplasty (UPPP; removal of tonsils and uvula and soft palate tissue). The most effective surgeries for OSA require multi-level treatment (palate and tongue base levels), are more complex, and are not as widely available since they require specialized surgical training and experience that are not generally available. In the literature, a successful surgical treatment is generally defined as a 50% reduction in AHI and an overall post-treatment AHI of < 20/hour^25^.

Surgical therapy is based in part on an anatomic assessment of the likely sites of obstruction. Anatomic analysis is most performed with fiberoptic examination of the upper airway, with radiographic imaging reserved for cases of suspected craniofacial abnormality. Under the classification of Fujita,^26,27^ patients with obstruction in the oropharynx only are considered type I; those with obstruction in the oropharynx and the hypopharynx are considered type II (mixed site of obstruction); while those with hypopharyngeal-only obstruction are considered type III. Most patients (>75%) have a mixed site of obstruction (type II)^28^. Multilevel obstruction is a common denominator for many patients with OSA, whether it is classified as mild, moderate, or severe disease^29-33^. Therefore, surgical treatments must be multi-level by necessity in order to address potential sites of obstruction. Whereas unselected single site surgery of the oropharynx (soft palate) with uvulopalatopharyngoplasty (UPPP) is successful only 40% of the time, surgical success can be improved to greater than 65% when UPPP is combined with procedures to address the base of tongue (hypopharynx). This 65% success rate of multi-level sleep surgery approaches the long-term adherence and success rate of CPAP therapy^25^.

Radiofrequency ablation (RFA) of the upper airway using FDA-cleared devices has demonstrated promise as a treatment alternative for obstructive sleep apnea in multiple published studies ^29-31, 34^. In these studies, repeated RFA of the soft palate and base of tongue region resulted in significant reductions in AHI and daytime sleepiness without significant complications. RFA has several advantages over traditional surgical approaches including its ability to address multiple levels of the airway (nose, palate, tongue); ability to perform in the office under local anesthesia; lower cost; and minimal pain and morbidity.

RFA enables the surgeon to direct the delivery of a specific amount of radiofrequency energy, measured in joules, to a specific site at a controlled temperature. This radiofrequency energy is delivered at relatively low power and voltage. Application of radiofrequency energy in this manner causes tissue ions to become agitated due to changes in electrical flow inherent in alternating current. These ionic shifts result in resistive heating by the tissue itself and in comparison to electrocautery the production of low temperatures (60° to 95°C). Protein, which denatures at temperatures in excess of 47°C, undergoes tissue coagulation along with surrounding stromal and vascular tissue. The lesion created by RFA is consistent with tissue coagulation and results in congestion, edema, and an acute inflammatory response within the first 24 hours. Over a period of 72 hours, the treated area progresses to tissue necrosis which may change to fibrotic tissue over the course of 10 days.

Volumetric reduction occurs in 2 stages. The contracted area of fibrosis occupies a smaller area than normal tissue and retracts the surrounding normal tissue resulting in the first stage of volumetric reduction of tissue. The second stage, resulting in further volumetric reduction, occurs over the course of several months as the body resorbs the area of fibrosis.

The CelonProSleep *plus*, the device used in this study, is FDA-cleared RFA device (K032838) that is indicated for ablation and coagulation of soft tissue in otorhinolaryngology (ENT) surgery including submucosal tissue shrinkage and tissue coagulation in the uvula/soft palate for the treatment of snoring. The system is intended for use by qualified medical personnel trained in the use of electrosurgical equipment.

## CelonProSleep plus

The CelonProSleep *plus* is a rigid, sterile, single-use, bipolar electrosurgical electrode. It is used in conjunction with the electrosurgical generator CelonLab ENT. The device is indicated for the ablation and coagulation of soft tissue in otorhinolaryngology surgery including submucosal tissue shrinkage and tissue coagulation in the uvula/soft palate for the treatment of snoring, and the soft palate (velopharynx) and base of tongue (oropharynx) for the treatment of mild to moderate obstructive sleep apnoea syndrome (OSAS).

Two electrodes located coaxially on the distal end of the device allow the generator to deliver a bipolar output to the tissue area, thus a neutral electrode or return conductor is not required. This is an advantage over other approved radiofrequency devices (ex. Somnoplasty) that require placement of a grounding pad on the patient and could interfere with implantable pacemakers. The power output on the device is controlled by the user on the generator unit. During its operation, tissue impedance is measured as coagulation status feedback. An acoustic signal from the generator unit informs the user that the coagulation process is complete and results in the automatic cessation of output power, ensuring safety in operation.

The dimensions of the CelonProSleep *plus* is designed for otorhinolaryngology surgeries—the bend angle and electrode length allow placement of electrode tip on the target tissue areas in the oral cavity and the trocar tip is appropriate for ablation and coagulation of tissues that are difficult to penetrate.

Fig. 2 -1 the CelonProSleep *plus*

Each electrode is supplied with an insulating cover that allowed exposure of only 1 cm of active electrode to avoid mucosal injury during treatment. The application time varied between four to six seconds per puncture (soft palate) and ten to sixteen seconds at the base of tongue, terminated by acoustic ‘end-indication’ and auto-stop facilitated by a thermistor and tissue impedance measurement at the probe tip. Energy delivery can be manually terminated by the operator if any pain or blanching of mucosa occurred.

## Safety of RF ablation of the soft palate and base of tongue

Radiofrequency ablation of the soft palate and base of tongue is considered as a minimally invasive therapy with much less side effects and complications in comparison to other surgical therapies for OSAS^39,^ ^40^.

Potential specific complications of radiofrequency ablation include bleeding, infection, ulceration, palatal fistula, tongue weakness, and taste disturbance alongside globus sensation. Long-term complications are unusual but globus sensation may persist in up to 10 % of patients. Most patients recover quickly however and report improvements in snoring although a recent systematic review indicated that further long-term studies are required^41-43^.

In a retrospective study, 130 patients underwent one to three sessions of RFA of soft palate (6 lesions) and base of tongue (6 lesions) with either Somnus (Gyros-ENT), or the CelonProSleep *plus* (Caroll et al. ^32^, with Dr. Gillespie, the PI in this study). The most common complication of upper airway RFA was mucosal ulceration (11%; mild side effect^45^), which healed with saline gargles within 10 days to 2 weeks. Ten patients (8%) had significant palatal or tongue edema (mild complication), which interfered with speech and/or swallowing but resolved within a few days of starting a taper dose of steroids. One patient had a temporary paresis of the lingual nerve (moderate complication), which resolved a couple of weeks after tongue base RFA. This was attributed to a pressure neuropathy from the laryngoscope because the lingual nerve was not within the field of the RFA application. No patient had a severe or prolonged dysphagia preventing oral diet immediately after the procedure. However, no information is given whether the complications caused by using the Somnus or the CelonProSleep *plus*^32^.

Factors that affect RFA complications may be repeated RFA treatment to the same tissue, learning curve of individual surgeons, energy delivered per lesion, temperature selection, anatomical site treated, and perioperative steroid use^45^.

Kezirian et al.^45^ reported incidence of complications after RF treatment of the soft palate and base of tongue. 22 publications on the soft palate with a total number of patients of 669 and 1406 RF treatment sessions were reviewed. The total number of complications was 119 (0.6%), with 111 minor, 7 moderate, and 1 major complication. Of the reviewed studies only one study used the CelonProSleep *plus*^67^ (Tatla et al., 2003: 10 patients, 20 treatment sessions, 120 lesions, 2 mild palatal swelling, mucosal ulceration in one lesion).

The review of 9 studies revealed an incidence of complications for the treatment of the base of tongue of 48 (2.7%). The number of patients in these studies was 614, and the number of treatment sessions was 1392. There were 10 minor, 28 moderate, and 10 major complications. None of these studies used the CelonProSleep *plus* RF device^45^.

Farrar et al., (2008, with Dr. Gillespie as PI)^35^ conducted a meta-analysis on published studies to obtain a precise estimate to the effectiveness of RFA in the treatment of OSA. The total number of soft palate complications was 13 (3.9% in 144 patients over 331 treatments sessions), and the total number of base of tongue complications was 38 (3.5% in 252 patients over 1092 treatment sessions). Out of 16 studies only one study used the CelonProSleep *plus* for RFA treatment of the base of tongue (den Herder^46^: 24 patients, 1-3 treatment sessions, 6 lesions/session, no complications).

Eight studies describe the use of the CelonProSleep *plus* for the RF treatment of the soft palate and base of tongue^32, 46, 62-67^. In seven of these studies there were 199 patients, and the number of lesions varied between 6 and 18 for the base of tongue, and 6 to 9 for the soft palate. The number of lesions among all studies was approximately a minimum of 2,400 (# of patient * # lesions * one session). There were 9 mild complications (0.37%, mucosal blanching), and one moderate complication (0.04%, tongue edema). Details on the eighth study, Carroll et al.^32^, and the number of complications are given above.

One of the factors that affect RFA complications is the amount of energy delivered per lesion^45^. A comparison between the studies in the above Table reveals that, the amount of applied energy by using the CelonProSleep *plus* RF device is much less than by using the Gyrus or Somnus devices. For one lesion in the base of tongue Gyrus/Somnus recommend target energy of 750 Joules (J). Celon recommends using a setting power of 7 W that due to the impedance-feedback auto-stop function results in an application time of 7.4 seconds. The applied energy with Celon RF device is E = P * t = 7 W * 7.4 s = 51.8 Joules. Thus, CelonProSleep *plus* applies only 51.8 J/lesion compared to the 750 J/lesion of Gyrus or Somnus.

The Somnus device has a higher energy input and is monopolar, exposing the entire body of the patient, whereas the CelonProSleep *plus* unit has inherent innovative bipolar tip safety. This ensures that only tissue in the immediate vicinity of the probe tip, which has a bipolar arrangement of electrodes in the needle, is exposed to the radiofrequency current. This removes some of the risks linked to the process and the need for a neutral electrode is lost, eliminating the risk of burns. Further safety is provided by acoustic feedback and an auto-stop power control, whereas the Somnus device relies on the operator visualizing the temperature and impedance signal.

Due to the bipolar electrode the CelonProSleep *plus* device coagulates much faster (although the power setting is less) than the Gyrus or Somnos systems. Therefore, less energy is needed to reach the coagulation threshold of the tissue. In the case of a very slow coagulation process, as happens with the Gyrus or Somnos devices, a big proportion of energy is transported away from the target region by circulating blood before the coagulation threshold is reached.

# Investigational device and methods

## Investigational device description

## 3.1.1 Intended use of the investigational device

The CelonProSleep *plus* is an FDA-cleared RFA device (K032838) that is indicated for ablation and coagulation of soft tissue in otorhinolaryngology (ENT) surgery including submucosal tissue shrinkage and tissue coagulation in the uvula/soft palate for the treatment of snoring.

### Any changes to the investigational device during the clinical investigation or any changes from the IB

No changes were implemented in the CelonProSleep plus bipolar coagulation electrode device during the clinical study.

## Clinical investigation plan (CIP)

### Objective

The primary effectiveness of the study was to demonstrate a clinically significant reduction of OSAS from Baseline PSG sleep study to the 6-Month follow-up PSG in adults (≥ 22 years) with obstructive sleep apnea (AHI 10-30) and BMI ≤ 32.

### Primary endpoint

The effectiveness of the CelonProSleep *plus* was assessed by demonstrating adequate reduction in AHI and ODI as defined below:

A responder to the CelonProSleep plus RFA treatment was defined as a patient with an AHI reduction ≥50% and a reduction of their ODI ≥25% at the 6-month follow-up PSG, and their AHI at the 6-month follow-up is ˂20.

### Secondary endpoints

The secondary endpoints were examined to provide additional support and details of the primary ‎endpoint findings concerning the RFA treatment. The secondary endpoints assessed important patient-based clinical outcomes to compliment the biophysiological primary outcomes. Secondary endpoints were evaluated using mean changes from baseline to 6 weeks post-treatment and to 6-months follow-up.

The following tests were used as secondary endpoint:

**Functional Outcomes Sleep Questionnaire (FOSQ)**

# The FOSQ is a validated instrument that assesses the effect of a subject’s daytime sleepiness on activities of ordinary living. It is a quality-of-life measure that is commonly used in clinical evaluation and management of OSA.

**Epworth Sleepiness Scale (ESS)**

The ESS is a validated instrument that rates a subject’s daytime sleepiness. It is a quality-of-life measure that is commonly used in clinical evaluation and management of OSA.

**Visual Analog Scale (VAS snoring)**

VAS is a validated instrument that rates different subjective parameters, including snoring. It is a quality-of-life measure that is commonly used in clinical evaluation and management of OSA. The VAS was completed by the subject’s bed partner.

**Bed partner questionnaire**

The Bed Partner questionnaire has four responses that describe the partner's opinion regarding snoring severity of the patient.

**e.** **Visual Analogue Scale (VAS) Drowsiness in the past week**

Snoring intensity is evaluated using a 100 cm visual analogue scale (VAS) from **0 to 100:** 0-9 represents no snoring, 10-39 represents minimally annoying snoring, 40-69 represents moderately annoying snoring, 70-90 represents annoying snoring and 91-100 represents extremely annoying snoring. The questionnaire represents scoring in the past week.

### Design

This study is a Multi-center, Open label, single arm, non-randomized study.

### Ethical considerations

The study was performed in accordance with Good Clinical Practices and recommendations guiding physicians in biomedical research involving human subjects adopted by the 18th World Medical Assembly, Helsinki, Finland, 1964 and later revisions.

The protocol and informed consent were reviewed and approved before enrollment of patients by the appropriate Institutional Review Board where the study was conducted.

### Data quality assurance

All required data for this trial was collected on standardized case report forms (CRFs) designed specifically for the study. The CRFs were completed by a trained clinical research coordinator at the investigational site. Worksheets were used to collect information that is not commonly recorded in medical records. Otherwise, all data was corroborated by clinic or hospital records. Source documents were available for review during monitoring visits. In the case of patient questionnaires, the CRF was completed by the subject or the subject completed a worksheet with the data then being transferred to a CRF by the clinical research coordinator, depending on the accepted practice at the site.

### Subject population

**Inclusion Criteria**

Patients who meet all of the following criteria may be given consideration for inclusion in this study:

1. Adults (≥ 22 years)
2. Self-report of daytime somnolence
3. Body mass index (BMI) ≤ 32
4. Mild to moderate obstructive sleep apnea (AHI 10-30; lowest O2 sat ≥ 80%) OSA based on a prior PSG conducted within 12 months of enrollment or based on a 2-night home sleep study using the WatchPAT 200S-3.
5. Evidence of palate and tongue base collapse on supine fiberoptic examination (Müller's maneuver)
6. Have failed or have not tolerated CPAP treatment (See Section 5.1for definitions)
7. Have been offered and are not interested in oral appliance therapy
8. No prior surgical treatment for OSAS other than nasal surgery or tonsillectomy.
9. Willing and capable of providing informed consent
10. Willing and capable to return for all follow-up visits and PSG sleep-studies and filling out the questionnaires.

**Exclusion Criteria**

1. Patients who meet any one of the following criteria will be excluded from this study:
2. Another significant sleep disorder (e.g., insomnia, periodic limb movement)
3. Absence of regular bedpartner
4. Tonsillar hypertrophy (3 or 4 plus)
5. Chronic Obstructive Pulmonary Disease (COPD)
6. Interstitial Lung Disease (ILD)
7. Cystic Fibrosis
8. Acute Respiratory Distress Syndrome (ARDS)
9. Nasal or supraglottic obstruction on fiberoptic examination
10. ASA class III, IV, V
11. Latex allergy
12. Lidocaine allergy
13. Pregnancy or plans to become pregnant
14. Note: women of childbearing potential must demonstrate a negative pregnancy test upon enrollment; those patients qualified to progress to RFA must also demonstrate a negative pregnancy test within 7 days prior to the date of RFA procedure.
15. Major depression or non-stabilized psychiatric disorder
16. Drug or alcohol abuse
17. Previous palatal or tongue surgery
18. Stable or unstable angina
19. CHF
20. Moderate or severe valvular disease
21. TIA/CVA
22. Carotid stenosis or endarterectomy
23. Anemia
24. Room air SpO2 < 95%
25. Pulmonary hypertension
26. Dialysis
27. Central or mixed apnea ≥ 10% of respiratory events
28. Participation in another clinical study (enrolled in any concurrent study) whose investigational plan is judged to interfere or affect any of the measures of this study

### Treatment and treatment allocation

During the study three treatment sessions of Radio Frequency Ablation (RFA) of the soft palate (7 lesions) and the base of tongue (6 lesions) were performed using the CelonProSleep *plus* device.

### RFA Treatment Schedule

RF treatments were performed as following:

- 1^st^ treatment - Visit 3 (week 6 of the study)
- 2^nd^ treatment – Visit 4 (week 12)
- 3^rd^ treatment – Visit 5 (week 18).

### Concomitant medications/treatments

Patients were provided with prescriptions for an antibiotic (amoxicillin or clindamycin), an oral steroid (7-day methylprednisolone taper pack), and an oral pain medication to take if needed (Acetaminophen Hydrocodone).

### Duration of follow-up

Study Follow-up duration was 6 months after the 3^rd^ RF treatment.

### Statistical design

#### Clinical investigation hypothesis or pass/fail criteria

The primary effectiveness of the study was to demonstrate a clinically significant reduction of OSAS from Baseline PSG sleep study to the 6-month follow-up PSG in adults (≥ 22 years) with obstructive sleep apnea (AHI 10-30) and BMI ≤ 32.

Effectiveness of the CelonProSleep *plus* was assessed by demonstrating adequate reduction in AHI and ODI as defined below:

A responder to the CelonProSleep *plus* RF treatment was defined as a patient with an AHI reduction ≥50% *and* a reduction of their ODI ≥25% at the 6-month follow-up PSG, and their AHI at the 6-month follow-up is ˂20.

The aim of the study was to show that at least 50% of participants met the primary endpoint above. This will be tested with one sided 97.5% confidence interval with ±20% margin of error.

In order to record and analyze data for determining the primary endpoints, patients underwent a PSG sleep study to establish a mean baseline AHI and ODI. Due to the approved study protocol, patients that already underwent PSG test up to 12 months before the screening could use the results of this test. Patients were then treated with a series of three treatments of upper airway radiofrequency ablation with the bipolar electrosurgical electrode CelonProSleep *plus* (Olympus Winter & Ibe, Germany) over 18 weeks. The patients underwent a second PSG sleep study at the final follow-up examination 6-months later. Mean changes in AHI were compared for each individual and among all patients. Surgical success is defined if at least 50% of participants experience at least 50% reduction in AHI and 25% reduction in ODI (Performance Goal, PG=50%).

A subject was counted as a non-responder (regardless of AHI measurement at 6 months) under the following circumstances:

a. Death (any cause) between treatment and the 6-month follow-up

b. Unsuccessful radiofrequency treatment attempt

c. Need for an alternative OSA treatment before the 6-month follow-up

d. Subjects who abandon the study

e. Subjects who exit the study after the last treatment, but before the 6-month follow-up.

#### Sample size calculation

The sample size proposed for this study was 48 patients. This sample size is comparable to that used in performance and safety studies using radiofrequency ablation for the treatment of upper airways (soft palate and base of tongue). In fact, considerable clinical data on the efficacy and safety RFA in OSAS is available from prior studies on this topic.

The following sample size considerations are based on the assumptions that performance goal (PG) =50% with 5% significance level and 80% power.

The sample size of n=43 +10% dropouts, i.e., n=48, is calculated by using the normal approximation for the z-score for testing inference about a single proportion p.

*H0: P_0_ < 50%*

*H1: P_1_ ≥ 50% and*

*P1-P0 ≥ 20%*

Below is the output of PASS12 NCSS program for sample size calculation:

| Analysis of One Proportion Tests  Numeric Results for testing H0: P = P0 versus H1: P > P0  Test Statistic: Z Test using S (P0)  Proportion  Given H0 Given H1 Difference Target Actual Reject H0  Power N (P0) (P1) (P1 - P0) Alpha Alpha Beta If Z ≥ This  α=0.025  0.8029 187 0.5000 0.6000 0.1000 0.0250 0.0285 0.1971 1.9600  0.8112 82 0.5000 0.6500 0.1500 0.0250 0.0299 0.1888 1.9600  0.8081 43 0.5000 0.7000 0.2000 0.0250 0.0330 0.1919 1.9600  0.81 26 0.5000 0.7500 0.2500 0.0250 0.0378 0.1805 1.9600 |
| --- |

Please note that the actual exact Alpha of 0.033 will meet the two-sided 5% significance.

#### Statistical Analysis Methods

Data was analyzed using the statistical software R: A Language and Environment for Statistical Computing, R Core Team, R Foundation for Statistical Computing, 2021 Version 4.05 2021.

Quantitative and qualitative data were collected. Descriptive statistics for all parameters were calculated. For continuous parameters descriptive statistics including mean, standard deviation, median, and range are reported. For ordinal parameters, counts and percentages are reported in addition to the mean, standard deviation, median, and range. Categorical parameters have counts and percentages reported.

Baseline demographics, medical-history and questionnaires-scores were analyzed using descriptive statistics.

The statistical analysis of the PSG data at baseline and follow-up visits were analyzed using paired t-test and linear-by-linear χ2 tests for the AHI-Levels.

Comparison of Questionnaires’ scores (baseline ver. Follow-up visits) were tested using paired t-test. The graphical presentation includes the scatterplots overlaid by means and 95% CI. In addition, forest-plot is used to present the change after the 3rd RFA treatment and after the follow-up (6-months after 3rd RFA) for all questionnaires data together. Testing of the repeated measurements is carried out with mixed model with random intercept using nlme-package. Some data is missing, and the reported percentage is based on the available data, not on the overall complete case of n=43. All statistical tests were performed at significance level of 0.05, with no corrections for multiple testing. Evaluation of ODI and AHI and questionnaires association is done using scatter plot and correlation analysis. Heat-maps are generated to describe the pattern of associations and missing data pattern.

# Results

## Clinical Investigation Initiating date

The clinical investigation was initiated on February 2015, Medical University of South Carolina Charleston, SC

## Clinical Investigation completion date

The clinical investigation was completed in June 2021

**Study Centers**

Patients were recruited to the study at 6 study sites in the USA. Table 1 introduces the study sites.

| **Study Sites** | **Study completers** | **drop out** | **screening failure** | **Total** |
| --- | --- | --- | --- | --- |
| 1. Medical University South Carolina | 5 | 0 | 8 | **13** |
| 2. Blue Sleep Center, NY | 8 | 5 | 1 | **14** |
| 3. Sinus Center & Sleep Apnea Center, LA | 4 | 1 | 3 | **8** |
| 4. Associates of Otolaryngology, Denver | 4 | 1 | 1 | **6** |
| 5. Methodist HealthCare Fundation, Memphis | 3 | 0 | 3 | **6** |
| 6. ENT of Georgia, Atlanta | 19 | 3 | 1 | **23** |
| **Total.** | **43** | **10** | **17** | **70** |

*Table 4-1: Study medical sites*

## Disposition of subjects; number screened patients and patients who received therapy

The Flowchart in Figure 1 introduces patients’ disposition. The Flowchart shows that 17 patients were defined as screening failure, 43 patients completed the entire study course (Study Completers), and 10 patients were dropout or lost to follow-up (Study Dropouts). One patient (#2-4) was excluded from the efficacy statistical analysis as he showed extremely unreasonable AHI increased (11.4 at baseline to 31.5 at the last follow-up) as a result of machine failure. The patient refused to repeat the PSG. All dropout patients were included in the safety analysis.


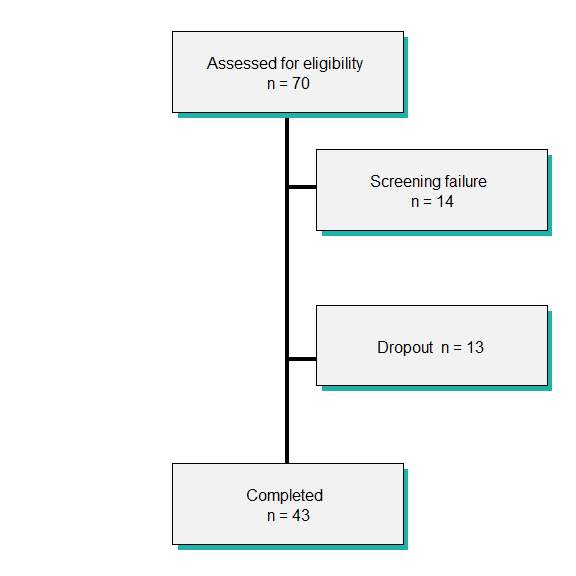


*Figure 4-1: Flow chart Diagram.*

## Subject demographics and other relevant baseline characteristics

Altogether, 70 patients were recruited to the study. Fourty three (43) patients completed the entire study course (Study Completers). Thirteen patients were drop-out (Study Dropouts): out of them, 12 patients lost to follow-up and one patient had unreasonable PSG results at the final PSG in follow-up due to device failure. As the patient refused to repeat the PSG he was defined as dropout.

Table 4-2 compares baseline demographics of Study Completers and Study Dropouts. The comparisons show no statistically significant differences between both groups. Tables 4-3 – 4-6 compare medical history (Table 4-3), CPAP history (Table 4-4), Sleep lab results (Table 4-5) and questionnaires at baseline (Table 4-6).

| **Baseline** | **Study Completers** | **Study Dropouts** | **p** |
| --- | --- | --- | --- |
| n | 43 | 13 |  |
| Gender (Male) (%) | 30 (71.4) | 11 (91.7) | 0.288 |
| Age (years) (mean (SD)) | 50.67 (11.20) | 53.75 (8.18) | 0.380 |
| Age (years) (median [Range]) | 51.00 [26.00, 72.00] | 54.00 [43.00, 71.00] | 0.397 |
| Race (%) |  |  | 0.232 |
| Afro-American | 4 (9.3) | 0 (0.0) |  |
| Caucasian | 31 (72.1) | 12 (100.0) |  |
| Hispanic | 2 (4.7) | 0 (0.0) |  |
| Other | 6 (14.0) | 0 (0.0) |  |
| BMI (mean (SD)) | 27.19 (3.72) | 26.00 (3.78) | 0.333 |
| BMI (median [Range]) | 27.40 [19.91, 32.70] | 25.46 [19.70, 33.80] | 0.254 |
| Gained weight recently (%) | 2 (4.7) | 1 (8.3) | 1.000 |
| Lost weight recently (%) | 1 (2.3) | 1 (9.1) | 0.868 |
| Exercise (%) |  |  | 0.140 |
| Daily | 5 (11.9) | 0 (0.0) |  |
| Frequently | 17 (40.5) | 5 (41.7) |  |
| Never | 0 (0.0) | 1 (8.3) |  |
| Occasionally | 18 (42.9) | 4 (33.3) |  |
| Rarely | 2 (4.8) | 2 (16.7) |  |

*Table 4-2. Patients’ demographic (Study Completers and Study Dropouts)*

**a. Medical history at Baseline**

| **Medical History** | **Study Completers** | **Study Dropouts** | **P** |
| --- | --- | --- | --- |
| n | 43 | 13 |  |
| Ever had a polysomnogram (sleep study) (%) | 40 (93.0) | 11 (91.7) | 0.999 |
| Hypertension (%) | 14 (32.6) | 4 (33.3) | 0.999 |
| Ischemic heart disease (%) | 0 (0.0) | 0 (0.0) | NA |
| Heart failure (%) | 0 (0.0) | 0 (0.0) | NA |
| Arrhythmia (%) | 1 (2.3) | 0 (0.0) | 0.999 |
| Stroke (%) | 0 (0.0) | 0 (0.0) | NA |
| Transient ischemic attack (%) | 0 (0.0) | 0 (0.0) | NA |
| Diabetes (%) | 3 (7.0) | 1 (8.3) | 0.999 |
| Hypercholesterolemia (%) | 7 (16.3) | 0 (0.0) | 0.314 |
| Renal failure (%) | 0 (0.0) | 0 (0.0) | NA |
| Peripheral vascular disease (%) | 0 (0.0) | 0 (0.0) | NA |
| Hepatic disease (%) | 0 (0.0) | 0 (0.0) | NA |
| Hematological disease (%) | 0 (0.0) | 0 (0.0) | NA |
| Sinusitis (%) | 2 (4.7) | 1 (8.3) | 0.999 |
| Thyroid disease (%) | 3 (7.0) | 0 (0.0) | 0.824 |
| Asthma (%) | 3 (7.0) | 0 (0.0) | 0.824 |
| Adenoidectomy (%) | 2 (4.7) | 0 (0.0) | 0.999 |
| Tonsillectomy (%) | 7 (16.3) | 1 (8.3) | 0.820 |
| Nasal septal surgery (%) | 7 (16.3) | 4 (33.3) | 0.369 |
| Maxillofacial (%) | 1 (2.3) | 0 (0.0) | 0.999 |
| LAUP (%) | 0 (0.0) | 0 (0.0) | NA |
| Uvulopalatoplasty (UPP) (%) | 0 (0.0) | 0 (0.0) | NA |
| Sinus surgery (%) | 5 (11.6) | 3 (25.0) | 0.485 |
| Tracheostomy (%) | 0 (0.0) | 0 (0.0) | NA |
| Allergies (%) | 18 (41.9) | 3 (25.0) | 0.467 |
| Smoking (%) | 3 (7.0) | 0 (0.0) | 0.824 |
| Alcohol abuse (%) | 3 (7.1) | 2 (16.7) | 0.661 |

*Table 4-3. Baseline Medical history of Study Completers and Study Dropouts*

**b. CPAP history at Baseline**

Table 4-4 introduces and compares baseline CPAP history of Study Completers and Study dropouts. Table 4-4 shows that most of the study participants (Study Completers and Study Dropouts) reported on CPAP intolerance (93% and 91.7% respectively) in addition to inability to use CPAP as indicated (35% and 41.7% respectively) and unwillingness (77% and 60% respectively). Statistical comparisons indicate no significant differences in the CPAP history of Study Completers and Study Dropouts.

| **CPAP history and AHI levels** | **Baseline Results** | | **p** |
| --- | --- | --- | --- |
|  | **Study Completers** | **Study Dropouts** |  |
| n | 43 | 13 |  |
| CPAP failure - indicated by inability to eliminate OSA (%)^a^ | 0 (0.0) | 0 (0.0) | NA |
| CPAP intolerance (%) | 40 (93.0) | 11 (91.7) | 0.999 |
| Inability to use CPAP as indicated (%)^b^ | 15 (34.9) | 5 (41.7) | 0.926 |
| Unwillingness to use CPAP (%)^c^ | 33 (76.7) | 7 (58.3) | 0.368 |
| Non-compliant with details for non-compliance (%) | 12 (27.9) | 4 (33.3) | 0.995 |
| Experiencing discomfort because CPAP pressure is too high (%) | 9 (20.9) | 3 (25.0) | 0.999 |
| Discomfort due to other reason (%) | 25 (58.1) | 6 (50.0) | 0.862 |
| Causes undesirable clinical effects (%) | 2 (4.7) | 2 (16.7) | 0.430 |
| Patient not using the system enough (%) | 3 (7.0) | 3 (25.0) | 0.212 |
| Patient symptoms persist despite CPAP use (%) | 3 (7.0) | 0 (0.0) | 0.824 |
| AHI level approximated by previous PSG (%) |  |  | 0.442 |
| 10-15 | 15 (34.9) | 2 (16.7) |  |
| 15-20 | 16 (37.2) | 5 (41.7) |  |
| 20-30 | 12 (27.9) | 5 (41.7) |  |
| ^a^ AHI remains greater than 10 despite CPAP usage | | | |
| ^b^ Device indicates less than 5 nights of usage per week (usage defined as more than 4 hours of use per night | | | |
| ^c^ Patient returns the CPAP system after attempting to use it | | | |

*Table 4-4. Baseline CPAP history of Study Completers and Study Dropouts.*

**c. AHI scores at Baseline**

Table 4-5 compares baseline AHI results (average) for Study Completers and Study Dropouts. The comparisons indicate similarity in baseline PSG results in both groups: AHI mean (19.6 (±7.1) and 19.15 (±6.93) respectively).

| **PSG Parameters** | **Baseline Results** | | **p** |
| --- | --- | --- | --- |
|  | **Study completers** | **Study Dropouts** |  |
| n | 43 | 13 |  |
| AHI (mean (SD)) | 19.70 (7.10) | 19.15 (6.93) | 0.839 |
| AHI (median [Range]) | 17.80 [10.40, 34.90] | 20.00 [10.40, 29.00] | 0.783 |
| Lowest O2 sat % (mean (SD)) | 84.28 (5.25) | 82.50 (3.09) | 0.269 |
| Lowest O2 sat % (median [Range]) | 84.00 [66.00, 96.00] | 82.00 [78.00, 87.00] | 0.116 |
| Mild-to-moderate OSA (%)^a^ | 41 (95.3) | 12 (100.0) | 1.000 |
| ^a^ AHI 10-30; Lowest O2 sat. > 80% | | | |

*Table 4-5. Baseline AHI results of Study Completers and Study Dropouts*

**d. ODI scores at Baseline**

Table 4-6 compares average scores of ODI at baseline and 6 months post-intervention. ODI mean was (12.8 (±7.7) and 12.15 (±10.18) respectively). Eleven patients did not have an ODI score at baseline as the ODI value could not be retrieved from the historical PSG sleep lab (up to one year before enrollment). Nevertheless, Statistical comparisons indicate no differences in the PSG baseline results in both groups.

| **PSG Parameters** | **Baseline Results** | | **p** |
| --- | --- | --- | --- |
|  | **Study completers** | **Study Dropouts** |  |
| n | 43 | 13 |  |
| ODI (mean (SD)) | 12.80 (7.74) | 12.15 (10.18) | 0.879 |
| ODI (median [Range]) | 11.65 [0.00, 31.20] | 8.80 [4.00, 27.00] | 0.650 |

*Table 4-6. Baseline PSG results of Study Completers and Study Dropouts*

**e. Questionnaires’ scores at Baseline**

Table 4-7 introduces and compares baseline scores of the study’ questionnaires. The scores of the questionnaires are presented as means and SD. Statistical comparisons indicate no significant differences in the various questionnaires between Study Completers and Study Dropouts.

| **Questionnaires** | **Baseline** | | **p** |
| --- | --- | --- | --- |
|  | **Study Completers** | **Study Dropouts** |  |
| n | 43 | 13 |  |
| Snoring Questionnaire (mean (SD)) | 5.33 (1.34) | 4.50 (1.31) | 0.071 |
| Bed Partner Questionnaire (mean (SD)) | 6.91 (2.20) | 6.88 (2.30) | 0.900 |
| Bed Partner Questionnaire (%) |  |  | 0.998 |
| (0-3) | 3 (7.0) | 1 (8.3) |  |
| (4-6) | 11 (25.6) | 3 (25.0) |  |
| (7-9) | 25 (58.1) | 7 (58.3) |  |
| 10 | 4 (9.3) | 1 (8.3) |  |
| Epworth Sleepiness Scale (ESS) (mean (SD)) | 11.19 (5.40) | 10.25 (2.99) | 0.524 |
| VAS during the past week (mean (SD))^a^ | 54.34 (26.33) | 55.92 (24.46) | 0.853 |
| SaO2 level (mean (SD))^b^ | 96.79 (1.63) | 96.45 (1.04) | 0.526 |
| FOSQ Total Score (mean (SD)) | 88.12 (20.16) | 95.58 (13.59) | 0.234 |
| ^a^ VAS range is alert (0) – drowsy (100) | | | |
| ^b^ SaO2 levels is % saturation | | | |

*Table 4-7 Baseline questionnaires results of Study Completers and Study Dropouts*

## CIP Compliance

This clinical investigation was conducted in accordance with the ethical principles that have their origin in the Declaration of Helsinki, Clinical investigation of medical devices for human subjects — ISO 14155:2011 Good Clinical Practice, and any regional or national regulations, as appropriate. The clinical investigation was initiated after all necessary IRB approvals were obtained from the appropriate regulatory authority in each clinical site.

All changes to the protocol were permitted after written approval from Olympus and the regulatory authorities.

## Results of the primary endpoints

According to the protocol, the primary efficacy objective of the study is to demonstrate a clinically significant reduction of OSAS post intervention compare to baseline, after 3 RFA treatments of the base of tongue and the soft palate in adults with mild to moderate obstructive sleep apnea (AHI 10-30).

The effectiveness of the RFA treatment performed with CelonProSleep *plus* was assessed by the following endpoints:

- AHI post-intervention ≤20
- AHI reduction ≥50% post intervention compared to baseline
- ODI reduction ≥25% post intervention compared to baseline; and
- Reduction of AHI ≥ 50% and ODI ≥ 25%) post-intervention.

Study success was defined as: at least 50% of study patients are defined as Responder.

AHI and ODI percentage’ change from baseline were calculated to obtain the rate of each endpoint as n/N, whereas n = number of participants experiencing the required AHI or ODI change and N = total eligible patients. 100*n/N is the percentage experiencing the required change in AHI or ODI. The statistical software used in the preparation of this report is NCSS 12 (Ver 12.0.2 2018). The following sections will introduce and discuss the results of each endpoint.

#### 4.**6.1 Analysis of AHI Results (AHI reduction)**

**AHI post-intervention ≤20**

Table 4-8 introduces the AHI levels at baseline and at 6 months post-intervention. The comparisons show that while at baseline 25 patients (58.1%) had AHI scores below 20, the vast majority of the patients (39 out of 43 = 90.7%) had AHI scores below 20 after the RFA treatment(s). Furthermore, while at baseline no patient had AHI scores below 5 (normal), 16 patients (**37.21%**) had AHI level below 5 at 6 months post-intervention, and thus, classified as normal. In addition, 18 patients (20.9%) had AHI scores above 20 at baseline, but only 4 patients (9.3%) have AHI scores above 20 after six months post-intervention. These results demonstrate the clinical benefits of the RFA treatment provided by the CelonProSleep *plus*. P-value for AHI levels is based on McNemar’s Chi-squared test.

|  | **Baseline**  **N (%)** | **Post Intervention**  **N (%)** |  |
| --- | --- | --- | --- |
| n | 43 | 43 |  |
| AHI level |  |  |  |
| **<5** | **0 (0)** | **16 (37.2)** |  |
| 5-15 | 16 (37.2) | 16 (37.2) |  |
| 15-20 | 9 (20.9) | 7 (16.3) |  |
| >20 | 18 (20.9) | 4 (9.3) |  |

*Table 4-8. AHI levels at baseline and 6 months post intervention, after 3^rd^ RFA treatment*

#### In clinical practice AHI score above 15 is associated with increased risk of cardiovascular disease and stroke. Therefore, the goal in clinical practice in to reduce the AHI below 15 and thus reducing the risk of cardiovascular diseases as well as reduction in snoring, improvement in sleep quality, and reduction in daytime sleepiness. In this study 32 patients reached an AHI level of 15 or below after the RFA treatment in comparison to 16 patients who had an AHI level of 15 or below at baseline (an increase of 100%).

**AHI reduction post-intervention compared to baseline**

Table 4-9 summarizes AHI average and median at baseline and at 6 months follow-up after the 3^rd^ RFA session.

The AHI reduction post-intervention was analyzed for Study Completers (n=43). As shown below, the RFA treatment, provided by the CelonProSleep *plus*, demonstrated statistically significant reduction in AHI (p<0.001) by comparing the average AHI at baseline to the AHI at six months follow-up. Out of 43 patients, 39 patients (90.7%) showed AHI reduction six months after the last RFA session. 23 patients (53.5%) showed AHI reduction greater than 50%. The comparisons of AHI scores are based on paired T-Test. Detailed Table that summarizes AHI scores of Study Completers is attached to this report as ANNEX 4. The reduction in AHI scores reveals the clinical benefit of the RFA treatments.

| **AHI** | **Baseline** | **Post Intervention** | **P** |
| --- | --- | --- | --- |
| n | 43 | 43 |  |
| AHI (mean (SD)) | 19.70 (7.10) | 9.86 (8.28) | ***<0.001*** |
| AHI (median [Range]) | 17.80 [10.40 - 34.90] | 7.5 [0.00 - 35.90] |  |

*Table 4-9. AHI results at baseline and at 6 months follow-up (after 3 RFA treatments)*

#### **4.6.2 Analysis of ODI Results (ODI reduction)**

Table 4-10 summarizes the ODI results at baseline and at 6 months after the 3^rd^ RFA treatment. As indicated above, eleven patients did not have an ODI score at baseline as the ODI value could not be retrieved from the historical PSG sleep study that was conducted up to one year before enrollment. The missing ODI scores are due to the fact that not every sleep lab calculated the ODI’ values, and the raw data was not available for the “off-line” calculations of ODI. Therefore, ODI levels at baseline were obtained from 32 patients only. As the study started at 2015, and as till recently ODI was not routinely used in clinical practice and wasn’t uniformly reported on PSG sleep studies performed for clinical reasons, the patients with an “old” PSG results didn’t have ODI scores. The recent reason for interest in ODI is the knowledge that hypoxia may be the primary driver of cardiovascular sequelae and not just lack of ventilation (apnea/hypopnea). Thus, ODI has become in recent years an important score in sleep studies.

In addition, 3 patients did not have the ODI scores at follow-up so that ODI scores at follow-up were obtained from 40 patients.

The ODI’ endpoint required a reduction of at least 25% in ODI score after FRA treatment. As shown below, the RFA treatment by the CelonProSleep *plus* demonstrated statistically significant reduction in ODI (p<0.005) while comparing average ODI at baseline to the 6 months follow-up, in which 21 patients (out of 32 = 65%) showed an ODI reduction of 25% and above. Furthermore, 16 patients (50%) showed an ODI reduction of 50% after RFA treatment. ODI comparisons are based on paired T-Test. Detailed Table that summarizes ODI score levels of Study Completers is attached to this report as ANNEX 4. The reduction of ODI in this study demonstrates the clinical benefit of the RFA treatment.

| **ODI** | **Baseline** | **6 Months Post Intervention** | **P** |
| --- | --- | --- | --- |
| n | 32* | 40** |  |
| ODI (mean (SD)) | 12.80 (7.74) | 8.79 (8.70) | ***0.005*** |
| ODI (median [Range]) | 11.65 [0.00 - 31.20] | 6.32 [0.00 - 30.40] | ***0.008*** |
| ** 11 patients did not have the ODI score at baseline as they used the results of former PSG test and the ODI score was not indicated.*  *** 3 patients did not have post-intervention ODI.* | | | |

*Table 4-10. ODI levels at baseline and 6 months post intervention, after 3^rd^ RFA treatment*

4.6.3 Summary of Endpoint Results

Study results indicated that:

1. **AHI score (<20) post-treatment** (at six months follow-up): **90.7%** of the patients, 39 out of 43, reached an AHI score smaller than 20 at the 6 months follow-up after the last RFA treatment. This result is well above the requirement that at least 50% of study patients will have their AHI score below 20. Thus, this endpoint is met.

2. **AHI scores reduction (≥50%) post-treatment:** **53.5%** of the patients, 23 out of 43, met this endpoint. This result is above the requirement that at least 50% of study patients will reduce their AHI in at least 50%. Thus, this endpoint is met.

3. **ODI reduction ≥25% post-treatment**: **65.67%** of the patients, 14 out of 32 patients who had ODI scores pre- and post- treatment, met this endpoint. This result is well above the requirement that at least 50% of study patients will reduce their ODI in at least 25%. Thus, this endpoint is met.

4. **AHI reduction ≥50% and ODI reduction ≥25%: 43.7% of the patients**, 14 out of 32 patients who had a full set of AHI and ODI scores, met this endpoint so that the combined endpoint(s)’ result is below the requirement that at least 50% of patients will meet this endpoint. This result is assumed to be due to a smaller group (32 patients) that had ODI scores at baseline. As mentioned above, patients were allowed to use PSG results within one year before participating in the study and eleven patients did not have the ODI results in the former PSG results. Furthermore - patients that already had PSG results refused to repeat the sleep lab.

The Table below (4-11) shows that over 50% of study patients met 3 out of 4 endpoints, while the 4^th^ endpoint (a combination of the AHI and the ODI reduction) is met by 43.7% of the patients (2 patients less than required to meet the success criterion).

| **Endpoints** | **Patients that met success criteria** | | **N** |
| --- | --- | --- | --- |
|  | **Rate n/N** | **Rate %** |  |
| 1. AHI ≤20 | 39/43 | ***90.7*** | 43 |
| 1. AHI reduction ≥50% | 23/43 | ***51.2*** | 43 |
| 1. ODI reduction ≥25% | 21/32 | ***65.6*** | 32 |
| 1. Combination of AHI reduction ≥50% and ODI reduction ≥25% in more than 50% of the patients | 14/32 | ***43.75*** | 32 |

*Table 4-11 summary of the analyses of endpoints’ results*

#### 4.**6.4 Analysis of OSA Results**

**Patients’ OSA severity at baseline and at six months follow-up**

The severity of sleep apnea was determined based on the AHI score (normal <5, mild sleep apnea 5-15, and moderate sleep apnea 15-30).

At baseline, no patients were classified as “normal” (AHI ≤5), 16 patients out of 43 (37.2%) were classified as “mild sleep apnea” (AHI 5-15), and 27 patients (62.8%) were classified as “moderate sleep apnea” (15≤ AHI ≤30)

Figures 4-2 and 4-3 below introduce sleep apnea severity (based on AHI scores) at 6 months follow-up compared to baseline. Figure 4-2 shows the AHI scores post-intervention for the patients defined as “mild sleep apnea” at baseline; Figure 4-3 shows the AHI scores post-intervention for the patients defined as “moderate sleep apnea” at baseline.

**Improvement of mild OSA patients**

After 3 sessions of RFA administrated to the base of tongue and the soft palate, four (4) patients out of 16 mild patients (25%) are defined as ***“normal”*** (AHI <5), thus gained a fully clinically improvement of their OSA symptoms. Further 10 patients are defined as mild patients after the treatment. The average AHI scores improved at follow-up compared to baseline (9±2.9 ver. 12.89±1.4 respectively). Statistical analysis of the AHI scores using Wilcoxon Singed Rank Test for Paired Data (using NCSS 12, 2018) reveals a statistically significant difference between AHI scores of the “mild” cases at baseline compared to post-treatment (p≤0.05). Thus, and although not defined as “normal”, the sleep quality of these patients was also improved due to the RFA treatment.

Two mild patients were defined as “moderate” at follow-up. The AHI scores of these two patients were 13.4 and 13.6 at baseline (upper range of the mild definition) and 19 and 18.2 (respectively) at follow-up.


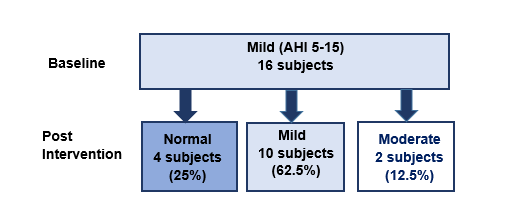


*Figure 4-2: Sleep apnea severity at baseline and at follow-up for patients defined as mild at baseline*

**Improvement of moderate OSA patients**

After 3 sessions of RFA administrated to the base of tongue and the soft palate, 12 patients out of 27 moderate patients (44.4%) were defined as ***“normal”*** (AHI <5) at follow-up, thus gained a fully clinically improvement of their OSA symptoms.

Additionally, further six patients out of 27 (22.2%) were defined as “mild” at follow-up demonstrating significant improvement in sleep quality after RFA treatment. Thus, **66.6%** of patients who were classified as “moderate” at baseline significantly improved their sleep quality and symptoms after RFA treatment.

Nine (“moderate”) patients were defined as “moderate” also at follow-up with no change in AHI score from 23.54±5.7 at baseline to 22.0±6.8 at follow-up.

Analysis of the AHI results at follow-up compared to baseline clearly indicates the clinical benefits of RFA treatment for sleep quality and symptomatic in patients suffering from OSAS. Most impressive is the improvement in sleep quality of the 12 patients (44.4%) who were classified as “moderate” at baseline and as “normal” after 3 RFA treatments.


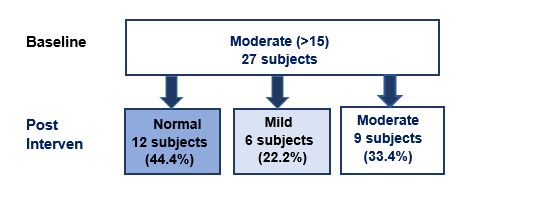


*Figure 4-3: Sleep apnea severity at baseline and at follow-up for patients defined moderate at baseline*

Table 4-12 summarizes patients’ distribution in accordance with OSA classification at baseline and at 6 months follow-up.

| **Sleep apnea categories** | **AHI Scores** | |
| --- | --- | --- |
|  | **Baseline**  **n=43** | **Post Intervention**  **n=43** |
| **Normal (<5)** | **0 subjects** | **16 subjects**  **(37.2%)** |
| **Mild (5-15)** | **16 Subjects**  **(37.2%)** | **16 subjects**  **(37.2%)** |
| **Moderate (15-30)** | **27 subjects**  **(62.8%)** | **11 Subjects**  **(25.6%)** |

*Table 4-12.* Patient’s classification before and after RFA treatment.

#### **4.6.5 Analysis of Questionnaires’ Results**

Altogether, 5 questionnaires were presented to study participants including one questionnaire addressing participant’s bed partner as following:

- Snoring questionnaire.
- Bed partner questionnaire.
- Epworth Sleepiness Scale (ESS)
- Functional Outcomes of Sleep Questionnaire (FOSQ)
- Visual Analogue Scale (VAS) Drowsiness in the past week

**Results of the snoring questionnaire**

This questionnaire includes eight questions where response options are – YES / NO as following:

1. Are you able to share a hotel room with a travel companion?  Yes  No

2. Has your companion ever moved to another room?  Yes  No

3. Do you snore while sleeping on your  back?  Stomach?

4. Difficulty breathing though your nose?  Yes  No

5. Mouth breathing at night (dry mouth in the morning)?  Yes  No

6. Excessive movements during sleep?  Yes  No

7. Wake up during the night with your heart pounding?  Yes  No

8. Any observed periods at night when you stop breathing?  Yes  No

For questions 2 and 4 to 8 a “No” answer means lack of snoring/sleep disorders, while a “Yes” answer indicates sleep disorders. For question 1 a “No” answer indicates sleep disorder, while a “Yes” answer indicates no sleep disorder. For question 3 any response (“back” or “stomach”) was regarded as sleep disorder, but if none of the options was marked the response was regarded no sleep disorder. For the statistical analyses a response that indicated sleep disorders was scored as “1”, while a response that indicated no sleep disorders was scored as “0”. Thus, a score of “7 - 8” means sever sleep disorders, a score of “4 – 6” moderate snoring, a score of “1 - 3” means mild sleep disorders and a score of “0” means no sleep disorder.

The average response at baseline was 5.3 (± 1.36) which indicates moderate snoring problems, whereas the snoring score at six weeks and six months follow-up were 3.68 (±1.33) and 3.45 (±1.62), respectively (mild sleep disorders). These results were found to be statistically significant from baseline to six-week after the 3^rd^ RFA treatment (p=0.01) and remained at significant level after six-month post-intervention (p=0.001). The results clearly demonstrate the improvement in the snoring condition following RFA treatments. Table 4-13 shows the results of the snoring questionnaire at baseline, 6 weeks after the 3^rd^ FRA treatment and at 6 months follow-up.

**Bed partner questionnaire**

The Bed Partner questionnaire has four responses that describe the partner's opinion regarding snoring severity of the patient:

1. (0-3) Occasional soft snoring; not bothersome to bed partner

2. (4-6) Persistent snoring; bothersome to bed partner

3. (7-9) Persistent loud snoring; frequently annoying bed partner

4. (10) Heroic snoring; continuous; loud snoring not tolerated by bed partner

Bed partner score range is 0 to10, while 0-3 means no snoring problem; 4-6 mild snoring; 7-9 moderate snoring and 10 represent severe snoring disturbance. In order to summarize the results, mid values were used (an answer of 0-3 was calculated as 1.5, an answer of 4-6 was calculated as 5, an answer of 7-9 was calculated as 8 and an answer of 10 was calculated as 10.

The average response at baseline was 7.0 (± 2.16) which indicates moderate snoring problems (loud snoring, frequently annoying bed partner). At six weeks and six months after the last RFA treatment the average scores were 3.21 (±2.38) and 3.12 (±2.38), respectively (a level which is not bothersome to bed partner). These results were statistically significant from baseline to six-week after the 3^rd^ RFA treatment (p =0.001) and remained the same after 6 months follow-up. Furthermore, at baseline only 4 bed partners (9.3%) classified their partner as “not bothersome to bed partner” (0-3), whereas at 6-weeks and 6-month follow-ups most bed partners (61% and 63.4% respectively) classified their partners as “not bothersome to bed partner” (0-3). In addition, the vast majority of bed partners (67.44%) considered the partner’s snoring as moderate (7-9, frequently annoying bed partner) and severe (10, loud snoring, not tolerated by bed partner), but only 3 bed partners (7.3%) considered the snoring as moderate, and one patient (2.3%) as severe at the 6-weeks and 6-month follow-up. These results clearly demonstrate the clinical benefit of the RFA treatment provided by the CelonProSleep *plus* as reflected by bed partner’s opinion. Table 4-13 shows the results of the Bed Partner questionnaire at baseline, 6 weeks and 6 months follow-up visits.

**Epworth Sleepiness Scale (ESS)**

The ESS is a self-administered questionnaire with eight questions. On a 4-point scale (0-3), patients were asked to rate their usual chances of dozing off or falling asleep while engaged in eight different activities. The ESS score (the sum of 8 item scores, 0-3) can range from 0 to 24. The higher the ESS score, the higher that person’s average sleep propensity in daily life (ASP), or their ‘daytime sleepiness’. In general, ESS final score can be interpreted as follows:

#### 0-5 Lower Normal Daytime Sleepiness

#### 6-10 Higher Normal Daytime Sleepiness

#### 11-12 Mild Excessive Daytime Sleepiness

#### 13-15 Moderate Excessive Daytime Sleepiness

#### 16-24 Severe Excessive Daytime Sleepiness.

The average ESS score at baseline was 11.19 (± 5.40) which indicates mild excessive daytime sleepiness and was reduced at six weeks and six months post-intervention to 5.59 (±3.22) and 5.95 (±3.51), respectively. Both results indicate lower normal daytime sleepiness. The results of this questionnaire show significant improvement from mild excessive daylight sleepiness or falling asleep at baseline to lower normal daytime sleepiness at 6-weeks and 6-months follow-up. These results clearly demonstrate the clinical benefit of the RFA treatment provided by the CelonProSleep *plus* as reflected by the ESS questionnaire. The results were found to be statistically significant (p-0.001). Table 4-13 shows the results of the ESS questionnaire at baseline, 6 weeks and 6 months follow-up visits.

**Functional Outcomes of Sleep Questionnaire (FOSQ)**

The Functional Outcomes of Sleep Questionnaire (FOSQ) is the gold-standard, disease-specific instrument designed to assess the impact of sleepiness on the ability to conduct daily activities Six subscales are represented in the questionnaire:

- General Productivity - 8 questions
- Social Outcome – 2 questions
- Activity Level – 9 questions
- Vigilance – 7 questions
- Intimate Relationships and Sexual Activity – 4 questions

Subscale Scores: A response score of 0 for an item is coded as N/A or a missing response. The potential range of scores for any item is I - 4. Total Score is calculated by the mean of the subscale scores multiply by the number of questions for which there is a score. The potential range of scores for the Total Score is 5 -20 where higher score indicate higher activity level.

The average FOSQ score at baseline is 14.91 which indicates mild excessive daytime sleepiness. At six weeks and six months post-intervention the FOSQ scores were 16.96 and 17.41 respectively which indicate better functional status following RFA treatment.

Table 4 – 13 introduces the FOSQ scores at baseline, 6-weeks, and 6-months post-intervention. These results were found to be statistically significant (p-0.001).

**VAS – Drowsiness in the past week**

Snoring intensity is evaluated using a 100 mm visual analogue scale (VAS) from **0 to 100:** 0-9 represents no snoring, 10-39 represents minimally annoying snoring, 40-69 represents moderately annoying snoring, 70-90 represents annoying snoring and 91-100 represents extremely annoying snoring. The questionnaire represents scoring in the past week.

The average VAS Snoring score at baseline was 54.34 (± 26.33) which indicates moderate annoying snoring. At six weeks and six months post-intervention the VAS Snoring scores were 34.92 (±24.98) and 31.08 (±24.72, respectively) which indicates normal minimally annoying snoring. Thus, these results of the VAS - drowsiness show improvement from moderate annoying snoring at baseline to minimally annoying snoring at 6-weeks and 6-months post-intervention. These results, as in other questionnaires, demonstrate the clinical benefit of the RFA treatment provided by the CelonProSleep *plus*. The results are statistically significant (p-0.001) and presented in Table 4-11.

| **Questionnaires** | **Baseline Average** | **6 weeks post intervention** | **6 months post intervention** | **P.val: a vs b** | **P.val: a vs c** |
| --- | --- | --- | --- | --- | --- |
| Snoring Questionnaire | 5.33 ±1.34 | 3.68±1.33 | 3.41 ±1.66 | 0.013 | 0.001 |
| Bed Partner Questionnaire (BPQ) | 7 ± 2.16 | 3.21 ± 2.38 | 3.12 ±2.38 | <0.001 | <0.001 |
| Epworth Sleepiness Scale (ESS) | 11.19 ± 5.40 | 5.59 ±3.22 | 5.95 ± 3.51 | <0.001 | <0.001 |
| Functional Outcomes of Sleep Questionnaire (FOSQ) | 14.91 | 16.96 | 17.41 | <0.01 | <0.001 |
| VAS: Drowsiness in the past week | 54.34 ± 26.33 | 34.92 ± 24.98 | 31.08 ± 24.72 | <0.05 | <0.001 |
| Note: | | | | | |
| - Values are Mean ± STDEV | | | | | |
| - P is based on Paired-T test | | | | | |
| - ESS: range 0-24 general level of daytime sleepiness | | | | | |
| - FOSQ: range 5-20 general level functionality burden | | | | | |
| - VAS range is alert (0) – drowsy (100) in the past week | | | | | |

*Table 4-13. Pairwise comparisons of questionnaires at baseline vs. 6 weeks and 6 months follow-up*

#### **4.6.6 Evaluation of pain level and recovery rate after RFA treatments**

Pain level was documented using the VAS scale (0-10, where “0” is no pain and “10” is unbearable pain). Pain level measurements were performed directly after each RFA treatment and 1, 3, 7, and 10 days after each RFA treatment. The average pain level directly after RFA treatment was low (2.78 ±2.41). A sharp decrease in the pain level was documented at three days following treatment (1.1±1.1), and the level of pain continued to decline on subsequent visits as well. The low pain level and reduction in pain level were observed after each treatment. These results indicate that the RFA treatment involves minimal level of pain directly after the RFA treatment, which declines almost completely 3 days after the treatment.

| **RFA Treatment** | **Pain Level** | | | | |
| --- | --- | --- | --- | --- | --- |
|  | Directly after treatment  (Mean ±STDEV) | 1 day after treatment  (Mean ±STDEV) | 3 days after treatment  (Mean ±STDEV) | 7 days after treatment  (Mean ±STDEV) | 10 days after treatment  (Mean ±STDEV) |
| 1^st^ RFA treatment | 2.78 ±2.41 | 2.76 ±1.59 | 1.1±1.14 | 0.32 ± 0.75 | 0.23 ± 0.76 |
| 2^nd^ RFA treatment | 2.88 ±1.73 | 2.34 ±1.7 | 0.69 ± 0.84 | 0.38 ± 0.88 | 0.24 ± 0.76 |
| 3^rd^ RFA treatment | 2.83 ± 1.62 | 2.57 ± 1.65 | 1.02 ±1.07 | 0.54 ± 1.05 | 0.63 ± 1.5 |

*Table 4-14: pain level directly after the RFA treatment and 1, 3, 7, and 10 days later*

*Figure 4-2: pain level after each RFA treatment*

In addition, the vast majority of the patients reported complete recovery of the soft palate and the tongue base (95% and 97% respectively) at six weeks after the RFA treatment and 100% at six months follow-up. These results clearly indicate that the RFA treatments performed with CelonProSleep *plus* are safe, fast recovery, and almost painless.

| **Treated Area** | **Recovery Rate** | |
| --- | --- | --- |
|  | **Six weeks after last**  **RFA treatment** | **Six months after last**  **RFA treatment** |
| n | 43 | 43 |
| Soft palate (%) | 38 (95.0) | 43 (100.0) |
| Tongue base (%) | 39 (97.5) | 43 (100.0) |

*Table 4-15. Healing Evaluation at 6 weeks and 6 months after 3rd RFA treatment*

#### **4.6.7 Summary of all adverse events**

No serious adverse events (SAEs) were detected or reported during the study. Eleven mild adverse events (AEs) were reported in five patients, whereas 8 of the 11 AEs (64%) occurred in 2 out of the 5 patients. Out of the 5 patients, one patient was a dropout, and the other four patients completed the entire course of the study. Six AEs were solved without any treatment, and 5 AEs were treated with saline or antibiotics. Two patients were administrated steroids. As the first trial in the United States to examine the use of Celon ProSleep *plus* for the base of tongue region, it is notable that there were no adverse events related to base of tongue abscess; bleeding/hematoma; airway obstruction; change in taste; or prolonged dysphagia. Detailed table that summarizes the adverse events is attached to this report as ANNEX 2.

#### **4.6.8 Adverse device effects**

There were no device deficiencies during the study.

#### 4**.6.9 Any needed subgroup analyses for special populations (i.e., gender, racial/cultural/ethnic subgroups), as appropriate**

No subgroup analysis was needed.

#### **An accountability of all subjects with a description of how missing data or deviation(s) were dealt with in the analysis, including subjects**

Subjects who met the eligibility criteria were enrolled to the study. Subject enrollment began in November 2014, and the last subject was enrolled in January 2020. A total of 70 subjects were enrolled in the study. Fourteen (14) subjects were screening failure, and 13 subjects were lost to follow-up. Thus, 43 subjects completed the study course. Database lock for the clinical study was in October 2021. As shown in Table 4-16 below, patient accountability was observed throughout the study. At final follow up visit, (6 months after the last RFA treatment), subject accountability was approximately 76.8%, demonstrating good study compliance. All study visits were completed at the time of database lock.

Table 4-16: Subject Accountability

|  | **Visit 1 Screening Visit** | **Visit 2**  **Baseline** | **Visit 3**  **1^st^ RFA Treatment** | **Visit 4**  **2^nd^ RFA Treatment** | **Visit 5**  **3^rd^ RFA Treatment** | **Visit 6**  **Follow-up (6 weeks post 3^rd^ treatment)** | **Visit 7**  **Final Follow-up** | **Visit 8**  **Final Study Visit** |
| --- | --- | --- | --- | --- | --- | --- | --- | --- |
| Theoretical | 70 | 60 | 56 | 56 | 51 | 49 | 45 | 45 |
| Deaths (Cumulative) | 0 | 0 | 0 | 0 | 0 | 0 | 0 | 0 |
| No show |  |  |  |  |  | 1 |  |  |
| Withdraw or lost to follow-up (Cumulative) |  |  |  | 5 | 7 | 9 | 11 | 13 |
| Screening failure | 3 | 10 | 14 | 0 | 0 | 0 | 0 | 0 |
| Expected* | 67 | 60 | 56 | 56 | 51 | 49 | 47 | 45 |
| Actual ** | 67 | 60 | 56 | 51 | 49 | 46 | 45 | 43 |
| % Follow Up *** | 100% | 100% | 100% | 91.1% | 96.1% | 87.5% | 80.3% | 76.8% |

* Expected equals Theoretical minus Not Yet Overdue minus Withdrawals

** Actual equals’ subjects who attended the specific visit (not missing).

*** % Follow up equals Actual divided by Expected times 100

Below is a detailed description of screening failure and withdraw/lost to follow-up cases:

Visit 1: Patient: **05-07,** patient did not arrive for screening visit; patients **05-13, 5-14** baseline AHI was too low, no data for screening visit is available.

Visit 2: Patients **1-2, 1-3, 1-4, 1-5, 1-6, 1-9, 1-10** performed Visit 1 but were screening failures and did not perform Visit 2

Visit 3: Patients **1-1, 3-1, 3-2** and **4-1** left after Visit 2. They were excluded and considered as screening failures

Visit 4: Patients **2-3, 2-6, 3-9, 6-11** and **6-15** didn’t show up after Visit 3. They were considered as lost to follow up as they either didn’t answer to phone calls or moved away.

Visit 5: Patients **2-9** and **6-7** did not want to continue with the study after Visit 4.

Visit 6: Patients **3-5** and **4- 7** stopped after Visit 5 without giving any reason.

Visit 7: Patients **2-11** and **6-22** did not want to continue the study after Visit 6, the reason is not known.

Visit 8: Patients **2-4** (after V8) and **2-7** (after V7) were excluded (after Visit 8 and Visit 7, respectively) due to “unclear PSG” which was recorded as a protocol deviation: “PSG equipment replacement part needed”.

#### 4.6.11 **Listings of deaths and reasons for deaths**

No death occurred throughout the study.

# 5 Discussion and overall conclusions

**Clinical performance, effectiveness, or safety results and any other endpoints**

This clinical study was performed in order to evaluate the safety and effectiveness of three RFA treatments performed using CelonProSleep *plus* in OSAS patients. Safety was assessed by rate of serious adverse events or adverse events during the study, pain level and healing speed after treatments. Efficacy was evaluated by level of improvement in OSAS markers: AHI and ODI improvements after 3 RFA treatments compared to baseline. In addition, patients and bed partners were asked to fill out questionnaires at baseline, after each treatment and at 6-months follow-up.

## Safety

The RFA treatment is a minimally invasive treatment. No serious adverse events were detected or reported during the study. Eleven adverse events were reported in five patients, most of them (8 AEs, 64%) occurred in two patients. One patient was a dropout, and the other four patients completed the entire course of the study. All adverse events were mild. Out of 11 AEs six cases were resolved without any treatment, 5 cases were treated with saline or antibiotics and two patients were administrated with steroids. As the first trial in the United States to examine the use of Celon ProSleep plus for the base of tongue region, it is notable that there were no adverse events related to base of tongue abscess; bleeding/hematoma; airway obstruction; change in taste; or prolonged dysphagia.

Pain level was documented using the VAS scale (0-10, where 0 is no pain and 10 is unbearable pain). Pain level measurements were performed directly after the RFA treatment and 1, 3, 7, and 10 days after each RFA treatment. The average pain level directly after RFA treatment was low (2.78 ±2.41). A sharp decrease in the pain level was documented at three days following treatment (1.1±1.1), and the level of pain continued to decline on subsequent visits as well. The low pain level and reduction in pain level were observed after each treatment. These results indicate that the RFA treatment involves minimal level of pain directly after the RFA treatment, which declines almost completely 3 days after the treatment.

## Efficacy of the RFA Treatment

The effectiveness of the RFA treatment performed with CelonProSleep *plus* was assessed by the following endpoints:

- AHI post intervention ≤20.
- AHI reduction ≥50% after RFA treatment compared to baseline.
- ODI reduction ≥25% after RFA treatment compared to baseline; and
- AHI Reduction ≥ 50% and ODI reduction ≥ 25%) after treatment compared to baseline.

Study success was defined as: at least 50% of study patients are defined Responder.

Study results indicate that:

1. **AHI score (<20) post-treatment** (at six months follow-up): **90.7%** of the patients, 39 out of 43, reached an AHI score smaller than 20 at 6 months follow-up after the last RFA treatment. This result is well above the requirement that at least 50% of study patients will have their AHI score below 20. Thus, this endpoint is met.

2. **AHI scores reduction (≥50%) post-treatment:** **53.5%** of the patients, 23 out of 43, met this endpoint. This result is above the requirement that at least 50% of study patients will reduce their AHI in at least 50%. Thus, this endpoint is met.

3. **ODI reduction ≥25% post-treatment**: **65.67%** of the patients, 14 out of 32 patients who had ODI scores pre- and post- treatment, met this endpoint. This result is well above the requirement that at least 50% of study patients will reduce their ODI in at least 25%. Thus, this endpoint is met.

4. **AHI reduction ≥50% and ODI reduction ≥25%: 43.7% of the patients**, 14 out of 32 patients who had a full set of AHI and ODI scores, met this endpoint so that the combined endpoint(s)’ result is slightly below the requirement that at least 50% of patients will meet this endpoint. This result is assumed to be due to a smaller group (32 patients) that had ODI scores at baseline. As mentioned above, patients were allowed to use PSG results within one year before participating in the study and eleven patients did not have the ODI results in the former PSG results. Furthermore - patients that already had PSG results refused to repeat the sleep lab.

Endpoint analyses show that over 50% of study patients met 3 out of 4 endpoints, while the 4^th^ endpoint (AHI reduction ≥50% and ODI reduction ≥25%) was met by 43.7% of the patients (2 patients below requirement to meet the success criterion).

**Study duration**

The study lasted 6.5 years. In this long period 56 patients were recruited to the study, and only 43 (out of 56 -76.7%) completed the entire study course. This long study duration indicates the trouble in recruiting subjects. Patients found the study procedure too complicated with too many study visits (altogether 14 visits) and in addition two sleep lab visits. This is also the reason for a large percentage of dropouts (24%). The difficulty to recruit and maintain the patients in the study is a factor that affects the results of the study. Nevertheless – study results do indicate the significant impact of the RFA treatments on the sleep quality of study patients.

## Clinical effect of RFA treatments on OSAS patients

Another way to examine the impact of the RFA treatments done by the CelonProSleep *plus* in OSAS patients is to evaluate the change in sleep quality post-intervention compared to baseline. The sleep quality was determined based on the AHI scores (healthy <5, mild sleep apnea 5-15, and moderate sleep apnea 15≤ AHI ≤30).

Whereas at baseline no patient was classified as "normal" (AHI ≤5), 16 patients out of 43 (37.2%) had AHI <5 after the RFA treatment and are classified as "normal". Furthermore, whereas at baseline most patients (27 = 62.8%) were classified as having "moderate sleep apnea" (15≤ AHI ≤30), only 11 patients (25.6%) had 15≤ AHI ≤30 at the end of the study.

The AHI results post-intervention reveal the significant *clinical benefit* of the RFA treatments on sleep quality. Most impressive is the improvement in sleep quality in 12 patients (44.4%) who were classified as "moderate" at baseline but as "normal" after three RFA treatments. In addition, four patients (25%) who were classified as "mild" at baseline were classified as "normal" after the RFA treatments. Figure 4-3 introduce the transition in classification of study subjects before and after RFA treatments.

Fig. 4-3 OSA status of patients before and after RFA treatment


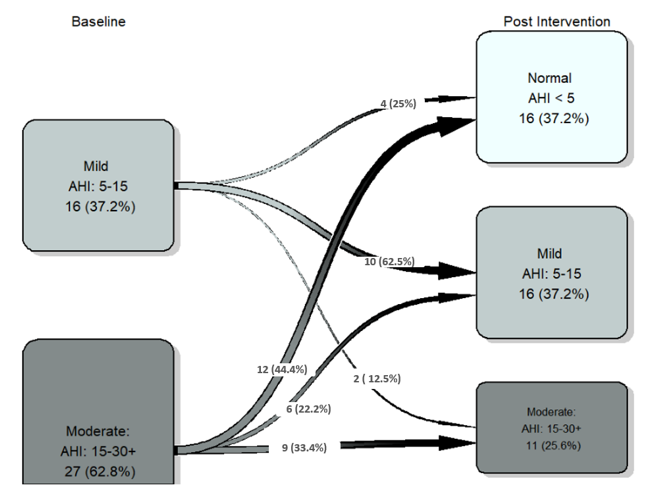


## Questionnaire results

Altogether, five questionnaires were presented to study participants, including one questionnaire addressing the participant's bed partner as follows:

- Snoring questionnaire
- Bed partner questionnaire
- Epworth Sleepiness Scale (ESS)
- Functional Outcomes of Sleep Questionnaire (FOSQ)
- Visual Analogue Scale (VAS) Drowsiness in the past week

The analyses of the results of all questionnaires indicate improvement in snoring, daytime sleepiness, level of activity, and drowsiness of the patients after RFA treatment. The difference between baseline scores, post-intervention and at follow-up scores are all statistically significant. These results reveal the improvement of the RFA treatments on different daily life aspects of OSA patients.

The results of these questionnaires indicate that the RFA treatments, provided by the CelonProSleep *plus,* significantly improves the sleep quality of OSA patients and level of their activities while reducing daytime drowsiness and OSA symptoms.

In summary the RFA treatment is minimally invasive, the level of risk associated with this treatment is very low with a low pain level and no serious adverse events (SAEs). Study results indicate significant clinical improvement in sleep quality and symptomatic of study patients.

## Assessment of risks and benefits

**Benefits:**

- The RFA treatments are minimally invasive treatments.
- The treatment is a short and simple procedure that can be performed in physician office.
- The treatment avoids the risk to the airway required by general anesthesia for other sleep surgical procedures.
- The procedure is multi-level and able to address the two main sites of airway collapsibility in the soft palate and tongue.
- Study results indicated that the RFA treatment is effective in improvement of sleep quality resulting in a higher level of daytime activities and reduction of daytime drowsiness.

**Risks**

- The RFA treatment is associated with low level of pain.
- Side effects that were reported:
- Uvula swelling, making speech, and swallowing uncomfortable,
- Palatal and uvular edema
- Painful mucosal ulceration on patient’s tongue.

All side effects were either declined without treatment or were resolved within a few days after medication. As the first U.S. trial of CelonProSleep *plus* for the base of tongue, it is notable that there were no serious adverse effects associated with its use in this anatomical region.

In summary – the RFA treatments are minimally invasive, short in time and simple procedure that improve very effectively OSA symptomatic resulting in improved sleep- and life quality with a very low risk level.

### 5.5.1 Any specific benefits or special precautions required for individual subjects or groups considered to be at risk

Study results indicated that the RFA treatment is safe and beneficial to OSAS patients.

### 5.5.2 Any limitations of the clinical investigation including but not limited to:

***Selection, retention, and compliance of subjects***

Out of 56 patients, 13 patients (23%) were lost to follow-up during the study (5 patients lost to FU after the first RFA treatment; 2 patients lost to FU after the second RFA treatment; 2 patients lost to FU after the 3^rd^ RFA treatment; and 2 patients lost to follow-up at each visit 6, 7 and 8). As these patients didn’t complete the study course, their last PSG test is missing and therefore we were not able to compare AHI and ODI scores before and after treatment. However, the PSG results retrieved from the patients that completed the study showed the improvement of AHI and ODI scores that was achieved by the RFA treatments.

#### 5.5.3 **Selection, retention, adherence (to CIP, instructions for use and the requirements of this document) of investigation sites and users, and investigation site environment type(s)**

All investigators adhered to the study protocol.

#### 5.5.4 Bias introduced by missing observations, by confounders and by 1) and 2) above

Eleven patients (out of 43) did not have an ODI score at baseline as the ODI value could not be retrieved from the historical PSG sleep lab (up to one year before enrollment). Therefore, ODI scores at baseline were obtained from 32 patients. It should be noted that ODI is not routinely used in clinical practice and therefore is not uniformly reported on sleep tests performed for clinical reasons.

# 6 Abbreviated terms and definitions

| RF | Radio Frequency |
| --- | --- |
| RFA | Radio Frequency Ablation |
| OSAS | Obstructive Sleep Apnea Syndrome |
| OSA | Obstructive Sleep Apnea |
| AHI | apnea-hypopnea index |
| BMI | Body mass index |
| PSG | polysomnography |
| UPPP | uvulopalatopharyngoplasty - removal of tonsils and uvula and soft palate tissue) |
| CPAP | Continuous positive airway pressure |
| COPD | Chronic Obstructive Pulmonary Disease |
| ILD | Interstitial Lung Disease |
| ARDS | Acute Respiratory Distress Syndrome |
| AASM | American Academy of Sleep Medicine |
| ASA | Physical Status Classification System |
| ODI | Oxygen desaturation index |
| FOSQ | Functional Outcomes Sleep Questionnaire |
| ESS | Epworth Sleepiness Scale |
| PM | portable monitor­ing |
| VAS | Visual Analog Scale |
| AE | Adverse Event |
| ADE | Adverse Device Effect |
| UADE | Unanticipated Adverse Device Effect |
| SADE | Serious Adverse Device Effect |
| TIA | transient ischemic attack |
| CVA | Cerebrovascular accident |
| CHF | Congestive heart failure |
| CA | Competent Authority |
| CCMO | Central Committee on Research Involving Human Subjects |
| GCP | Good Clinical Practice |
| ICF | Informed Consent Form |
| (S)AE | (Serious) Adverse Event |
| SUSAR | Suspected Unexpected Serious Adverse Reaction |
| WMO | Medical Research Involving Human Subjects Act |
| iCTE | Insufflated containment tissue extraction technique (iCTE) |
| FDA | Food and Drug Administration |
| IRB | Institutional Review Board |

# 7 Ethics

**7.1** Confirmation that the clinical investigation was conducted in accordance with the ethical principles in the Declaration of Helsinki

The Clinical Investigation was conducted in accordance with the Ethical Principles as laid down in the Declaration of Helsinki and with ISO-14155:2020.

### Statement that informed consent was obtained and when it was obtained

Informed consent was obtained from every patient enrolled into this Clinical Investigation prior to any study related procedure.

# 8 Investigators and administrative structure of clinical investigation

### A list of investigators, including their affiliations

|  | Investigator | Study site |
| --- | --- | --- |
| 1. | Prof. M. Boyd Gillespie (CCI) | Medical University South Carolina, SC |
| 2. | Dr. Keith A. Swartz, MD | Otolaryngology, Denver Colorado |
| 3. | Prof. M. Boyd Gillespie | Methodist Healthcare Foundation, Memphis, TN |
| 4. | Dr. Jordan Stern | Blue Sleep Center, NY |
| 5. | Dr. David M Alessi | Alessi Clinic, Beverly Hills, CA |
| 6. | Dr. Howard Herman M.D. | ENT of Georgia, Atlanta, Georgia |

### Names and addresses of any external organizations (such as core laboratories, CROs, consultants or other contractors) that contributed to the clinical investigation

1. Qsite Medical (CRO). Haavoda 31, Bynyamina 30500, Israel

2. IntegriStat (Statistician): Brodezki 31, Tel Aviv 69051, Israel

# Annexes to the report

### Annex 1 Instructions for use

***Caution:*** *The bipolar coagulation electrode is intended for use by qualified medical personnel trained in the use of electrosurgical equipment and targeted puncture of areas of organs. Please also observe Section 1 (Safety and Warnings) and also the user manuals for the power control unit and the accessories*.

**Preparation**

1. Follow the general guidelines for anesthesia, disinfection and working under sterile ambient conditions.

2. Check the sterile instrument packaging for damage and remove the bipolar coagulation electrode (Do not use coagulation electrodes from damaged sterile packaging!).

***Caution:*** *Check that all the sterile packaging are undamaged. After removing the instruments and accessories, check them for damage. Do not use damaged products or products from damaged sterile packaging.*

3. Remove the protective tube from the tip of the bipolar coagulation electrode.

4. Check the bipolar coagulation electrode, cable and connector for damage (Do not use damaged accessories!).

5. Insert the connector into the outlet socket of the power control unit.

**Setting the power level**

The recommended power setting for the CelonProSleep *plus* bipolar coagulation electrode is **10-13 watts** in the soft palate. The exposure time is automatically controlled by the power control unit and typically ranges from 2-10 seconds. The power setting can be individually adapted according to the physician's assessment of the clinical findings.

***Caution:*** *When using bipolar radiofrequency induced thermotherapy (RFITT), a lower power level may lead to longer application times and therefore a larger coagulation volume. This could result in damage to the mucosa.*

**Treatment steps**

1. Insert the electrode tip into the depth of tissue until the plastic shaft tube contacts the tissue. Both electrodes and part of the shaft must be completely in the tissue.

***Caution:*** *The bipolar coagulation electrode is designed for use in soft tissue only. Incorrect use, e.g., inserting into hard tissue or bending the electrode, may cause mechanical damage to the electrode tip. The bipolar coagulation electrode should be checked for damage after each puncture. Never use a defective bipolar coagulation electrode*.

***Caution:*** *Proper placement of the bipolar coagulation electrode is essential. Improper placement of the electrode could result in damage to the mucosa.*

2. Activate the power output by depressing the footswitch and hold the footswitch down until the treatment has been completed.

3. End the power output manually at any time, or when the end of the procedure is indicated (acoustic signal), by releasing the footswitch.

4. Remove the bipolar coagulation electrode from the tissue.

5. Repeat the procedure at different positions based upon the clinical judgment of the physician

6. Follow the general guidelines for wound care in otorhinolaryngological procedures.

#### ANNEX 2 Adverse Events

Adverse events reported 11 times from 5 participants, see tables below. One Ae was reported by Site 4 as a throat pain. All other 10 reports were from 4 patients at site 5. The Adverse events occurred at the RFA site and were procedure related. It was noted that all events were either resolved or improved at the follow-up visit. This is unfortunate, since these patients who suffered the adverse events had also failed to improve AHI and thus failed to benefit from the procedure. Two patients from site 5 reported multiple adverse events which all appeared to be related to the same burden.

| ID | Adverse Event | Outcome |
| --- | --- | --- |
| 4-6 | Throat pain | Resolved |
| 5-10 | On POD2 patient noted continued dysphagia that has persisted POD3. Patient noted that his symptoms have been improving. | improved |
| 5-4 | Patient has swelling of tongue base and uvula starting morning after REA1. This is causing her pain with speech and swallowing. She has noted that after day two this swelling and pain has begun to improve. | Improved |
| 5-4 | On post-op day one patient woke up with uvula swelling that is making speech and swallowing uncomfortable. She reports that it is not as significant as the first time this happened. | Persisting |
| 5-4 | Patient developed palatal and uvular edema, will continue steroid as planned. She also developed a mucosal ulceration. We instructed her on the use of saline gurgle to treat this. | Resolved |
| 5-4 | On post-op day 3 patient has mild mucosal ulceration. We instructed her in the use of saline gargle to treat this. | Resolved |
| 5-5 | Patient developed increasing pain in mouth starting on 22.06 and had low grade fever associated with her pain. She noted to start her pain medication again to tolerate soft foods. She has developed a fissure in the middle of her tongue which has a mild infection. | Improved |
| 5-6 | On POD1 patient developed uvula edema and palatal edema. It is causing discomfort during speech, swallowing and supine sleep. | Resolved |
| 5-6 | On POD 3 patient confirmed to have palatal edema and developed mucosal ulceration. She was encouraged to continue her steroid therapy and to start saline gurgles as tolerated. | Improved |
| 5-6 | On POD 10 patient confirmed to have mild palatal edema and mucosal ulceration. These all appear to be improving. She was encouraged to continue saline gurgles as tolerated. | Improved |
| 5-6 | Patient arrived for 10-Day post RFAIII visit and it was noticed on exam that there was a painful mucosal ulceration on her tongue. | Improved |

Table 1: Adverse events

**ANNEX 3** **Patients AHI and ODI variables**

| **Patient id** | **status** | **AHI BL** | **ODI BL** | **AHI FU** | **ODI FU** |
| --- | --- | --- | --- | --- | --- |
| 1-7 | completed | 21.4 |  | 16.2 | 14.06 |
| 1-8 | completed | 10.4 | 9.84 | 4.2 | 3.64 |
| 1-11 | completed | 19.6 |  | 10.1 | 6.44 |
| 1-12 | completed | 30.0 |  | 28.2 | 30.30 |
| 1-13 | completed | 14.0 |  | 6.6 | 8.60 |
| 2-1 | completed | 16.5 | 5.60 | 2.8 | 1.90 |
| 2-2 | completed | 17.0 | 18.40 | 16.9 | 13.10 |
| 2-3 | drop out | 19.8 |  |  |  |
| 2-4 | drop out | 11.4 | 9.60 | 31.5 | 9.50 |
| 2-5 | completed | 12.3 | 4.40 | 5.1 | 1.30 |
| 2-6 | drop out | 20.2 |  |  |  |
| 2-7 | drop out |  |  |  |  |
| 2-8 | completed | 29.3 | 27.50 | 1.3 | 1.80 |
| 2-9 | drop out | 28.0 |  |  |  |
| 2-10 | completed | 13.8 | 11.20 | 10.7 | 6.90 |
| 2-11 | drop out | 27.7 |  |  |  |
| 2-12 | completed | 14.1 | 8.90 | 8.6 | 4.20 |
| 2-13 | completed | 18.2 | 17.30 | 4.7 | 2.20 |
| 2-14 | completed | 14.9 | 10.20 | 0.3 | 2.10 |
| 2-16 | completed | 23.3 | 13.40 | 1.7 | 1.40 |
| 3-5 | drop out |  |  |  |  |
| 3-6 | completed | 15.9 |  | 12.7 | 10.70 |
| 3-7 | completed | 18.2 |  | 10.3 |  |
| 3-8 | completed | 16.4 |  | 7.0 |  |
| 3-9 | drop out | 16.5 |  |  |  |
| 3-10 | completed | 30.6 |  | 35.9 |  |
| 4-2 | completed | 17.8 | 18.10 | 18.5 | 25.80 |
| 4-3 | completed | 11.0 | 14.50 | 10.2 | 9.60 |
| 4-4 | completed | 13.4 | 15.90 | 19.0 | 17.90 |
| 4-6 | completed | 13.6 | 12.50 | 18.2 | 6.20 |
| 4-7 | drop out | 12.1 |  |  |  |
| 5-4 | completed | 16.4 | 17.10 | 19.0 | 27.60 |
| 5-6 | completed | 11.4 | 7.30 | 12.9 | 7.50 |
| 5-10 | completed | 23.8 | 31.20 | 25.5 | 30.40 |
| 6-1 | completed | 14.2 | 0.00 | 5.9 | 2.00 |
| 6-2 | completed | 23.9 |  | 3.5 | 5.00 |
| 6-3 | completed | 33.1 | 13.00 | 4.1 | 24.00 |
| 6-4 | completed | 20.4 |  | 1.6 | 1.00 |
| 6-5 | completed | 20.6 |  | 4.6 | 8.00 |
| 6-6 | completed | 25.6 | 12.00 | 7.5 | 16.00 |
| 6-7 | drop out | 29.0 |  |  |  |
| 6-8 | completed | 29.7 | 3.90 | 0.0 | 1.00 |
| 6-9 | completed | 33.8 | 27.00 | 3.2 | 18.00 |
| 6-10 | completed | 13.1 | 7.00 | 0.7 | 2.00 |
| 6-11 | drop out | 10.4 | 4.00 |  |  |
| 6-12 | completed | 24.5 | 9.00 | 11.3 | 11.00 |
| 6-13 | completed | 30.0 | 11.30 | 23.0 | 0.00 |
| 6-14 | completed | 24.9 | 24.00 | 15.1 | 10.00 |
| 6-15 | drop out |  | 27.00 |  |  |
| 6-16 | completed | 34.9 | 22.00 | 1.9 | 1.00 |
| 6-17 | completed | 24.5 | 18.00 | 1.1 | 1.00 |
| 6-18 | completed | 11.5 | 4.00 | 10.4 | 7.00 |
| 6-19 | completed | 11.9 | 2.00 | 13.2 | 5.00 |
| 6-20 | completed | 12.6 | 7.00 | 3.9 | 3.00 |
| 6-21 | completed | 14.7 | 6.00 | 6.4 | 3.00 |
| 6-22 | drop out | 21.7 | 8.00 |  |  |
| Note: BL- Baseline; FU – 6 months follow-up, after 3^rd^ treatment. | | | | | |
|  | | | | | |
|  | | | | | |

ANNEX 4 Study Protocol

REFERENCES

1. Fletcher EC, et al. Undiagnosed sleep apnea in patients with essential hypertension. Ann Intern Med 1985;103:190-195.
2. Millman RP, et al. Daytime hypertension in obstructive sleep apnea: prevalence and contributing risk factors. Chest 1991;99:861-866.
3. Partinen M. and Palomaki H. Snoring and cerebral infarction. Lancet 1985;2:1325-1326.
4. Koskenvuo M, et al. S. Snoring as a risk factor for ischaemic heart disease and stroke in men. BMJ 1987;294:16-19.
5. Hung J, et al. Association of sleep apnoea with myocardial infarction in men. Lancet 1990;336:261-264.
6. Malone S, et al. Obstructive sleep apnoea in patients with dilated cardiomyopathy: effects of continuous positive airway pressure. Lancet 1991;338:1480-1484.
7. Malone S, et al. Obstructive sleep apnoea in patients with dilated cardiomyopathy: effects of continuous positive airway pressure. Lancet 1991;338:1480-1484.
8. Waldhorn RE, et al. Long-term compliance with nasal continuous positive airway pressure therapy of obstructive sleep apnea. Chest. 1990;97:33-38.
9. Meurice JC, et al. Predictive factors of long-term compliance with nasal continuous positive airway pressure treatment in sleep apnea syndrome. Chest. 1994;105:429-433.
10. Rauscher H, et al. Nasal CPAP and weight loss in hypertensive patients with obstructive sleep apnea. Thorax. 1993;48:529-533.
11. Grunstein RR. Sleep-related breathing disorders, 5: nasal continuous positive airway pressure treatment for obstructive sleep apnoea. Thorax. 1995;5;0:1106-1113.
12. Meurice JC, et al. Predictive factors of long-term compliance with nasal continuous positive airway pressure treatment in sleep apnea syndrome. Chest. 1994;105:429-433.
13. Kenny P, et al. Terris. A comparison of polysomnography and the WatchPAT 200S-3 in the diagnosis of obstructive sleep apnea. Otolaryngology–Head and Neck Surgery (2007) 137, 665-668.
14. American Academy of Sleep Medicine. Portable monitoring in the diagnosis of obstructive sleep apnea. J Clin Sleep Med 2006; 2:274.
15. Decision Memo for Continuous Positive Airway Pressure (CPAP) Therapy for Obstructive Sleep Apnea (OSA) (CAG-00093R). 2005. (Accessed at http://www.cms.hhs.gov/mcd/viewdecisionmemo. asp?id=110.)
16. Portable Monitoring Task Force of the American Academy of Sleep Medicine: [Collop NA](http://www.ncbi.nlm.nih.gov/pubmed?term=Collop%20NA%5BAuthor%5D&cauthor=true&cauthor_uid=18198809), et al. Clinical guidelines for the use of unattended portable monitors in the diagnosis of obstructive sleep apnea in adult patients. [J Clin Sleep Med.](http://www.ncbi.nlm.nih.gov/pubmed/18198809) 2007 Dec 15;3(7):737-47.
17. Decision Memo for Continuous Positive Airway Pressure (CPAP) Therapy for Obstructive Sleep Apnea (OSA) (CAG-00093R2). http://www.cms.gov/medicare-coverage-database/details/nca-decision-memo.aspx?NCAId=204&fromdb=true.
18. Ceylan T. et al. Quick Diagnosis in Obstructive Sleep Apnea Syndrome: WatchPAT-200. Iran Red Crescent Med J. 2012;**14**(8): 475-478
19. Pillar G, et al. Autonomic Arousal Index (AAI): An Automated Detection based on Peripheral Arterial Tonometry. Sleep 2002; 25(5):543-5
20. Pillar G, et al. An automatic ambulatory device for detection of AASM defined arousals from sleep: the WP100. Sleep Med 2003; 4(3):207-212.
21. Bar A, et al. Evaluation of a Portable Device Based on Peripheral Arterial Tone for Unattended Home Sleep Studies. Chest 2003; 123(3): 695-703.
22. Zou D, et al. Validation a Portable Monitoring Device for Sleep Apnea Diagnosis in a Population Based Cohort Using Synchronized Home Polysomnography. Sleep 2006; 29(3):367-374.
23. Ayas N, et al. Assessment of a Wrist-worn Device in the Detection of Obstructive Sleep Apnea. Sleep Med 2003; 4(5):435-442.
24. Pittman SD, et al. Using a Wrist-Worn Device Based on Peripheral Arterial Tonometry to Diagnose Obstructive Sleep Apnea: In-Laboratory and Ambulatory Validation. Sleep 2004; 27(5):923-933.
25. Sher AE, et al. The efficacy of surgical modification of the upper airway in adults with obstructive sleep apnea syndrome. Sleep 1996; 19: 156-177).
26. Fujita S. Pharyngeal surgery for obstructive sleep apnea and snoring. In: Fairbanks D, Fujita S, Ikematsu T, et al. Snoring and Obstructive Sleep Apnea. New York, NY; Raven Press; 1987:101-128.
27. Fujita S, et al. Evaluation of the effectiveness of uvulopalatopharyngoplasty. Laryngoscope 1985;95:70-74.
28. Ravesloot MJL and de Vries N. One hundred consecutive patients undergoing drug-induced sleep endoscopy: results and evaluation. Laryngoscope 2011; 121: 2710-2716.
29. Woodson BT, et al. A randomized trial of temperature-controlled radiofrequency, continuous positive airway pressure, and placebo for obstructive sleep apnea syndrome. Otolaryngol Head Neck Surg. 2003 Jun; 128(6):848-61.
30. Steward DL, et al. A comparison of radiofrequency treatment schemes for obstructive sleep apnea syndrome. Otolaryngol Head Neck Surg. 2004 May;130(5):579-85.
31. Riley RW, et al. An adjunctive method of radiofrequency volumetric tissue reduction of the tongue for OSAS. Otolaryngol Head Neck Surg. 2003 Jul;129(1):37-42.
32. Carroll W, et al. Snoring management with nasal surgery and upper airway radiofrequency ablation. Otolaryngol Head Neck Surg. 2012 Jun;146(6):1023-7. Epub 2012 Feb 8.
33. Ceylan K, et al. First-choice treatment in mild to moderate obstructive sleep apnea: single-stage, multilevel, temperature-controlled radiofrequency tissue volume reduction or nasal continuous positive airway pressure. Arch Otolaryngol Head Neck Surg. 2009 Sep;135(9):915-9.
34. Steward DL, et al. Multilevel temperature-controlled radiofrequency for obstructive sleep apnea: extended follow-up. Otolaryngol Head Neck Surg. 2005; 132: 630-5.
35. Farrar J, et al. Radiofrequency ablation for the treatment of obstructive sleep apnea: a meta-analysis. Laryngoscope 2008; 118: 1878-83.
36. Powell NB, et al. Radiofrequency volumetric reduction of the tongue: a porcine pilot study for the treatment of obstructive sleep apnea syndrome. Chest 1997; 111: 1348-55.
37. Friedman M, et al. Staging of obstructive sleep apnea/hypopnea syndrome: a guide to appropriate treatment. Laryngoscope 2004; 114: 354-359.
38. M Friedman. Sleep apnea and snoring: Surgical and non-surgical therapy. Sounders (Elsvier) 2009. (Muller’s maneuver).
39. Verse T, et al. Multilevel surgery for obstructive sleep apnea: short-term results. Otolaryngol Head Neck Surg. 2006; 134:571–7.
40. Stuck BA, et al. Complications of temperature-controlled radiofrequency volumetric tissue reduction for sleep-disordered breathing. Acta Otolaryngol 2003; 123:532–535.
41. Stuck BA. Radiofrequency-assisted uvulopalatoplasty for snoring: long-term follow-up. Laryngoscope 2009; 119:1617–1620.
42. Back LJ, et al. Radiofrequency ablation treatment of soft palate for patients with snoring: a systematic review of effectiveness and adverse effects. Laryngoscope 2009; 119:1241–1250.
43. Baisch A, et al. Combined radiofrequency assisted uvulopalatoplasty in the treatment of snoring. Eur Arch Otorhinolaryngol 2009; 266:125–130.
44. Kezirian EJ, Powell NB, Riley RW, et al. Incidence of complications in radiofrequency treatment of the upper airway. Laryngoscope 2005; 115: 1298–1304.
45. den Herder C, et al. Bipolar radiofrequency induced thermotherapy of the tongue base: Its complications, acceptance and effectiveness under local anesthesia. Eur Arch Otorhinolaryngol. 2006; 263: 1031–40.
46. Powell NB, et al. Radiofrequency tongue base reduction in sleep-disordered breathing: a pilot study. Otolaryngol Head Neck Surg 1999; 120: 656–664.
47. Nelson LM. Combined temperature-controlled radiofrequency tongue reduction and UPPP in apnea surgery. Ear Nose Throat J. 2001; 80:640–4.
48. Woodson BT, et al. A multi-institutional study of radiofrequency volumetric tissue reduction for OSAS. Otolaryngol Head Neck Surg 2001; 125: 303–311.
49. Stuck BA, et al. Tongue base reduction with temperature-controlled radiofrequency volumetric tissue reduction for treatment of obstructive sleep apnea syndrome. Acta Otolaryngol 2002; 122: 531–536.
50. Terris DJ, et al. Minimally invasive tongue base surgery for obstructive sleep apnoea. J Laryngol Otol 2002; 116: 716–721.
51. Li KK, et al. Temperature-controlled radiofrequency tongue base reduction for sleep-disordered breathing: Long-term outcomes. Otolaryngol Head Neck Surg. 2002; 127(3):230-4.
52. Fischer Y, et al. Multilevel temperature-controlled radiofrequency therapy of soft palate, base of tongue, and tonsils in adults with obstructive sleep apnea. Laryngoscope. 2003;113:1786–91.
53. Steward DL. Effectiveness of multilevel (tongue and palate) radiofrequency tissue ablation for patients with obstructive sleep apnea syndrome. Laryngoscope 2004; 114: 2073–2084.
54. Stuck BA, et al. Combined radiofrequency surgery of the tongue base and soft palate in obstructive sleep apnoea. Acta Otolaryngol. 2004;124:827–32.
55. Blumen M, et al. Radiofrequency ablation for the treatment of mild to moderate obstructive sleep apnea. Laryngoscope 2002; 112:2086–2092.
56. Holmlund T, et al. Effects of Radiofrequency versus Sham Surgery of the Soft Palate on Daytime Sleepiness. Laryngoscope. 2014 Jan 4. doi: 10.1002/lary.24580.
57. Fernández-Julián E, et al. Randomized study comparing two tongue base surgeries for moderate to severe obstructive sleep apnea syndrome. Otolaryngology–Head and Neck Surgery 2009; 140, 917-923.
58. Friedman M, et al. Minimally invasive single-stage multilevel treatment for obstructive sleep apnea/ hypopnea syndrome. Laryngoscope 2007; 117: 1859–1863.
59. Hultcranz E, et al. Long-term effects of radiofrequency ablation of the soft palate on snoring. Eur Arch Otorhinolaryngol. 2010; 267(1):137-42.
60. Pazos G, Mair EA. Complications of radiofrequency ablation in the treatment of sleep-disordered breathing. Otolaryngol Head Neck Surg 2001; 125: 462–466.
61. Balsevičius T, et al. Controlled trial of combined radiofrequency-assisted uvulopalatoplasty in the treatment of snoring and mild to moderate OSAS (pilot study). Sleep Breath. 2013; 17(2):695-703.
62. Civelek S, et al. GlideScope Video Laryngoscope–Assisted Tongue Base Radiofrequency for the Treatment of Obstructive Sleep Apnea: Pilot Study. Journal of Otolaryngology-Head & Neck Surgery 2010;39(4),329–334.
63. Aavan den Broek E, et al. UPPP combined w06ith radiofrequency thermotherapy of the tongue base for the treatment of obstructive sleep apnea syndrome. Eur Arch Otorhinolaryngol. 2008; 265(11):1361-5.
64. Heywood Rl, et al. Radiological airway changes following bipolar radiofrequency volumetric tissue reduction. The Journal of Laryngology & Otology (2010), 124, 1078–1084.
65. Olszewska E, et al. Selected surgical managements in snoring and obstructive sleep apnea patients. Med Sci Monit, 2012: 18(1): CR13-18.
66. Tatla T, et al. Celon (R) radiofrequency thermo-ablative palatoplasty for snoring—a pilot study. J Laryngol Otol 2003; 117:801–806.
67. Blumen M, et al. Radiofrequency tongue reduction through a cervical approach: a pilot study. Laryngoscope 2006; 116:1887–1893.
68. Jacobowitz O. Palatal and tongue base surgery for surgical treatment of obstructive sleep apnea: a prospective study. Otolaryngol Head Neck Surg. 2006; 135(2):258-264.
69. Walker et al. Extended follow-up of palatal implants for OSA treatment. Otolaryngol Head Neck Surg. 2007; 137(5):822-7.
70. Doff et al. Oral appliance versus continuous positive airway pressure in obstructive sleep apnea syndrome: a 2-year follow-up. Sleep. 2013 Sep 1;36(9):1289-96.
71. ZAR, Jerrold H. Biostatistical analysis (1999).
72. Joseph L. Fleiss, Bruce Levin, Myunghee Cho Paik. Statistical Methods for Rates & Proportions. 2003.
73. Newcombe, R.G. Interval estimations for the difference between independent proportions: Comparison of eleven methods. Statistics in Medicine 1998; 17, 873-890
74. Fugita S. Surgical treatment of obstructive sleep apnea: UPPP and lingualplasty (laser midline glossectomy). In: Guilleminault C, Partinen M, eds. Ostructive sleep apnea syndrome: clinical research and treatment. New York: Raven Press, 1990: 129-151.
75. Weaver TE et al. An instrument to measure functional status outcomes of excessive sleepiness. Sleep 1997; 20:835-843.
76. Grote L, et al. Therapy with nCPAP: incomplete elimination of sleep related breathing disorder. Eur Resp J 2000; 16:921-927.
77. Iber C, Ancoli-Israel S, Chesson A, Quan SF for the American Academy of Sleep Medicine. The AASM Manual for the Scoring of Sleep and Associated Events: Rules, Terminology and Technical Specifications, 1^st^ ed.: Westchester, Illinois: American Academy of Sleep Medicine, 2007.

1. 510(k) premarket notification numbers K102567, K042916, K010739 [↑](#footnote-ref-1)
